# Novel Split-Luciferase-Based Genetically Encoded Biosensors for Noninvasive Visualization of Rho GTPases

**DOI:** 10.1371/journal.pone.0062230

**Published:** 2013-04-16

**Authors:** Weibing Leng, Xiaohui Pang, Hongwei Xia, Mingxing Li, Liu Chen, Qiulin Tang, Dandan Yuan, Ronghui Li, Libo Li, Fabao Gao, Feng Bi

**Affiliations:** 1 Department of Medical Oncology, West China Hospital, Sichuan University, Chengdu, Sichuan, China; 2 Laboratory of Signal Transduction & Molecular Targeted Therapy, State Key Laboratory of Biotherapy, Sichuan University, Chengdu, Sichuan, China; 3 Department of Radiology, West China Hospital, Sichuan University, Chengdu, Sichuan, China; Karolinska Institutet, Sweden

## Abstract

Rho family GTPases are critical regulators of many important cellular processes and the dysregulation of their activities is implicated in a variety of human diseases including oncogenesis and propagation of malignancy. The traditional methods, such as “pull-down” or two-hybrid procedures, are poorly suited to dynamically evaluate the activity of Rho GTPases, especially in living mammalian cells. To provide a novel alternative approach to analyzing Rho GTPase-associated signaling pathways in vivo, we developed a series of bioluminescent biosensors based on the genetically engineered firefly luciferase. These split-luciferase-based biosensors enable non-invasive visualization and quantification of the activity of Rho GTPases in living subjects. The strategy is to reasonably split the gene of firefly luciferase protein into two inactive fragments and then respectively fuse the two fragments to Rho GTPase and the GTPase-binding domain (GBD) of the specific effector. Upon Rho GTPase interacting with the binding domain in a GTP-dependent manner, these two luciferase fragments are brought into close proximity, leading to luciferase reconstitution and photon production in the presence of the substrate. Using these bimolecular luminescence complementation (BiLC) biosensors, we successfully visualized and quantified the activities of the three best characterized Rho GTPases by measuring the luminescence in living cells. We also experimentally investigated the sensitivity of these Rho GTPase biosensors to upstream regulatory proteins and extracellular ligands without lysing cells and doing labor-intensive works. By virtue of the unique functional characteristics of bioluminescence imaging, the BiLC-based biosensors provide an enormous potential for in vivo imaging of Rho GTPase signaling pathways and high-throughput screening of therapeutic drugs targeted to Rho GTPases and (or) upstream molecules in the near future.

## Introduction

Rho GTPases constitute a large subfamily of the Ras superfamily and include several isofonns of CDC42, Rac and Rho. They function as intracellular molecular switches, cycling between a GDP-bond state (inactive) and a GTP-bound state (active). Rho GTPase signaling pathways regulate various cell biological processes [1]. The ability of GTPases to properly bind and hydrolyze GTP is an essential prerequisite for the maintenance of normal cellular function [2]. The switch between the GTP–GDP bond states is controlled by several accessory proteins: (1) the guanine nucleotide exchange factors (GEFs), which promote the exchange of GDP for GTP; (2) the GTPases-activating proteins (GAPs), which enhance the intrinsic GTPase activity; (3) the GDP dissociation inhibitors (GDIs), which significantly slow the rate of dissociation of GDP [3]. Various extracellular signals converge on Rho GTPases through a large numbers of GEFs and GAPs [4]. It is not surprising that the dysregulation of their activities can result in diverse diseases, including cancer, mental disabilities and neurological diseases [4], [5], [6]. Therefore, the Rho GTPase signaling pathway always is a research hotspot in many disciplines, with the clinical or preclinical goals of targeting them for molecular-targeted therapy of many diseases.

Molecular imaging, especially optical imaging, provides a new platform for noninvasive visualization of biological processes at molecular level in the whole organism. This technique bridges the gap between the identification of biomarkers and their clinical applications. Fluorescence marking techniques and fluorescence resonance energy transfer (FRET) analysis are being widely used in characterizing the spatiotemporal dynamics of Rho GTPases in living cells [7], [8]. Several strategies are employed to construct these biosensors. The most successful design is the unimolecular biosensors based on FRET, including the “Raichu” probes [9], [10] and other unimolecular probes [11], [12]. These biosensors, with high spatial and temoporal resolution, provide insight into the intricate networks. They may promise to resolve important uncertainties or seeming contradictory results in living cells.

Accumulating evidence indicates that Rho GTPases are involved in the formation and progression of tumors in vivo [13]. To advance our understanding of the pathophysiological function of Rho GTPase signaling pathways, it's necessary for us to extend our investigations from in vitro to in vivo [4], [14]. In addition, Rho GTPases and their associated proteins are potential therapeutic targets for cancer, cardiovascular disease and other diseases [15], [16], [17]. As a promising emerging technology, molecular imaging promotes the transformation of basic research into preclinical or clinical application. However,the FRET assay mentioned above suffer from some weaknesses, including the need for an external excitation source, low sensitivity, challenge for stable expression and autofluorescence [18]. These disadvantages potentially limit its usefulness in the whole organism and high-throughput screening (HTS) in drug development in the future. Therefore, the development of novel alternative biosensors, which are capable of ironing out these flaws, would provide complementary advantages for future preclinical applications.

Bioluminescence imaging, which harnesses the light-emitting reactions of enzymes such as luciferase by oxygenating a substrate molecule, is a sensitive imaging modality that enables in vivo analysis of cellular and molecular events. It offers important opportunities for investigating a vide variety of disease in intact animal models and systems [19] and provide an ideal tool for accelerating the evaluation of experimental therapeutic strategies [20]. Bioluminescence can circumvent cell and tissue auto-luminescence, which results in a better signal to noise ratio for bioluminescent assays [21]. These properties are just complementary to the disadvantages of the FRET assay. Here, based on bimolecular luminescence complementation (BiLC) strategy, we developed and characterized a series of novel bioluminescent biosensors for imaging the activities of Rho GTPases in live subjects. In this article, we describe the design, construction and characterizations of these BiLC-based biosensors. They provide a highly sensitive method for imaging Rho GTPase activity in living subjects and analyzing the dynamical responses to upstream regulatory proteins and extracellular factors in the pathways.

## Materials and Methods

### Ethics Statement

All experimental procedures with animals used in this study had been given prior approval by the Experimental Animal Manage Committee of Sichuan University under Contract 2011-0138472. Animal handling and all procedures on animals were carried out strictly according to the guidelines of the Animal Care and Use committee of Sichuan University and the Animal Ethics Committee Guidelines of the Animal Facility of the West China Hospital. The nude mice were maintained under specific pathogen free (SPF) conditions. Mice were gas anesthetized with isofluorane (2% isoflurane in 100% oxygen, 1 L/min) using the XGI-8 Gas Anesthesia Unit (Caliper Life Sciences) during all injection and imaging procedures.

### Chemicals, Enzymes and Reagents

Restriction and modification enzymes and DNA ligase were purchased from Fermentas (Thermo Fisher Scientific Inc. Waltham, USA). TransStart FastPfu DNA polymerase was obtained from TransGen Biotech (Beijing, CN). The plasmids pGL3-Basic and pRL-tk purchased from Promega (Madison, WI) were used as templates for the amplification of the luciferase fragments. All BiLC-based biosensors were constructed in a pcDNA3.1 (+) vector backbone (Invitrogen). Site-directed mutagenesis was performed using the QuickChange™ site-directed mutagenesis kit (Stratagene, Heidelberg, Germany). Plasmid extraction kits and DNA gel extraction kits were purchased from Qiagen (Valencia, CA). Lipofectamine 2000 transfection reagent was obtained from Invitrogen (Carlsbad, CA, USA). Bradykinin acetate salt (BK), insulin and lysophosphatidic acid (LPA) were purchased from Sigma-Aldrich (St. Louis, Missouri, USA). D-Luciferin potassium was from Xenogen (Alameda, CA). Coelenterazine was purchased from Regis (Morton Grove, IL, USA).

### Construction of Plasmids

**Figure 1:**
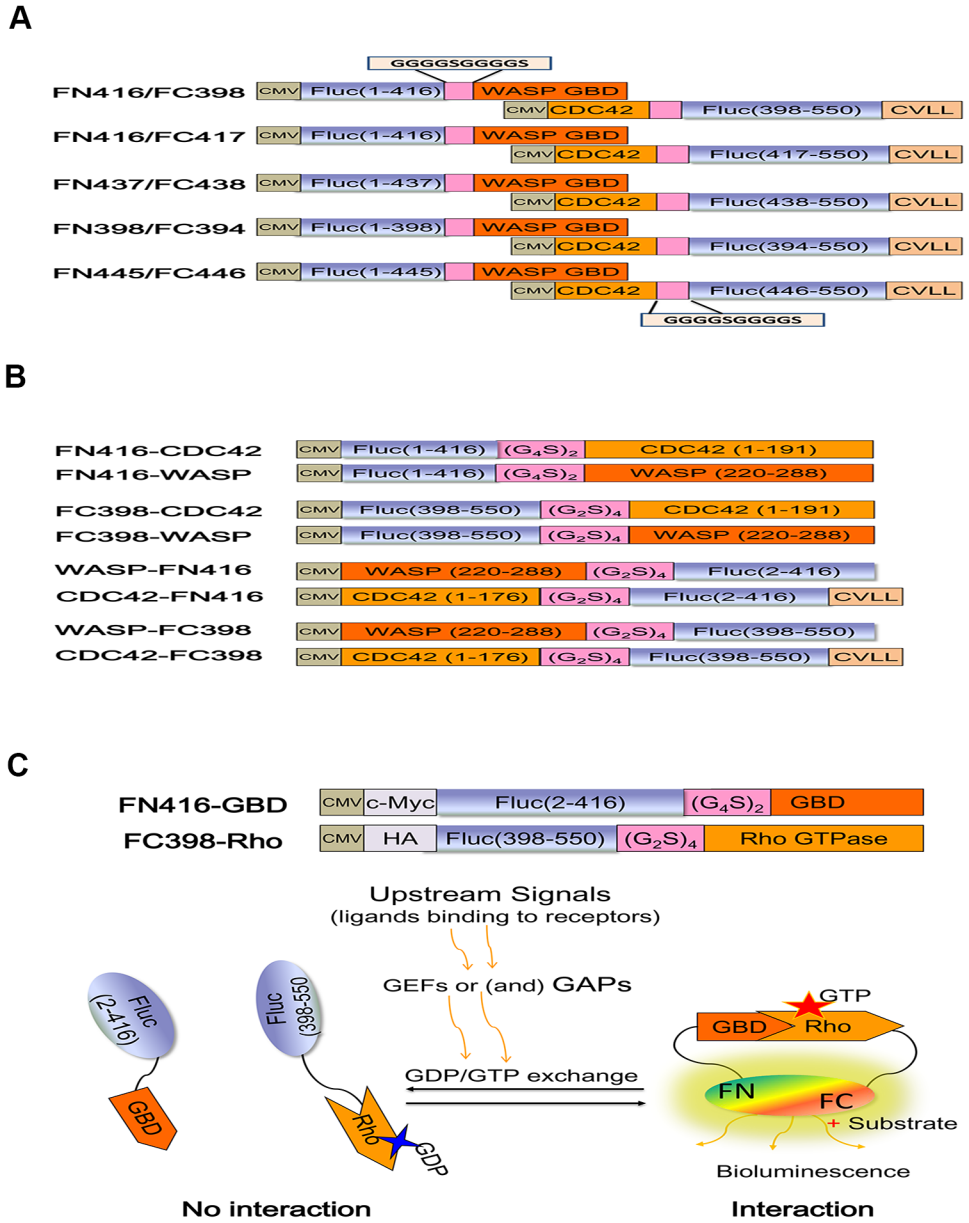
The schematic diagrams of the constructs and the mechanism for BiLC-based Rho GTPase biosensors. (A) The graphic schemes of the constructs used in process of optimizing the appropriate dissection sites of firefly luciferase. For the construct of CDC42-Cfluc, CDC42 (AA 1–176) was used and the carboxy-terminal region of CDC42 (AA171–191) was added to the downstream of the fusion protein, making sure the correct localization to the plasma membrane and the regulation of GDIs. (B) The graphic schemes of the constructs used in process of optimizing the appropriate orientation of the reporter fragments (FN and FC) and the interacting proteins (WASP and CDC42). If CDC42 was fused to the N-terminal of the reporter fragment, CDC42 (AA 1–176) was used and the carboxy-terminal region of CDC42 (AA 171–191) was added downstream of the reporter fragment. (C) The schematic diagram of the optimal configuration and the mechanism for BiLC-based Rho GTPase biosensors. We use the optimal configuration as representative to describe the mechanism of the biosensors. In this strategy, two non-functional luciferase fragments are respectively fused, in fame and with a short flexible linker (G_2_S)_2∼4_ or (G_4_S)_1∼2_, to Rho GTPase and the GBD of the specific effector. Once Rho GTPase is activated by upstream stimulating factors, the two luciferase fragments (luc1 and luc2) are brought into close proximity by the active Rho GTPase binding to the GBD of the effector, leading to the restoration of luciferase activity and photon production in presence of the substrate.

The renilla luciferase (RL) plasmid, which was cotransfected to normalize transfection efficiency, is pRL-tk (Promega, catalog E2241). The N and C portions of the firefly luciferase (FL) gene for each split point were amplified by PCR from pGL3-Basic (Promega, catalog E1751). The coding sequences of the Rho GTPases and their related proteins were amplified from the corresponding plasmid vectors kindly provided by Y. Zheng (University of Tennessee, USA). All the CDC42 biosensors were constructed by first generating the vectors pcDNA3.1-Nfluc and pcDNA3.1- Cfluc, following by insertion of PCR-amplified coding sequences of the interacting proteins (CDC42 and WASP GBD (AA 220–288)) in frame and with a short flexible linker (G_2_S)_2∼4_ or (G_4_S)_1∼2_ [22]. The frequently-used restriction enzyme sites in our experiment were NheI, HindIII, BamHI and XhoI. The combination pattern and orientation relationship between the luciferase fragments and the interacting proteins (CDC42 and WASP GBD) were changed by reasonably selecting these restriction enzyme sites. It's worth noting that if CDC42 was fused to the N-terminal of the reporter fragment, Cdc42 (AA 1–176) was used and the carboxy-terminal region of CDC42 (AA 171–191) was added downstream of the reporter fragment, imitating the design of “Raichu” biosensors [10]. This design is necessary for the correct localization to the plasma membrane and the regulation of GDIs [23]. If CDC42 was fused to the C-terminal, the full coding sequence of CDC42 was added downstream of the reporter fragment. The biosensors of other Rho GTPases (Rac1 and RhoA) were constructed using essentially the same procedure as was used to construct CDC42 biosensors based on the most optimal configuration (Nfluc416-effector/Cfluc398-Rho GTPase), which will be described in detail later. PAK GBD (AA 67–150) and PKN GBD (AA 13–112) were selected to specifically interact with Rac1 and RhoA, respectively. In order to perform coimmunoprecipitation experiments, c-myc epitope tag (EQKLISEEDL) and HA tag (YPYDVPDYA) were respectively added to the amino-terminal of Nflu416-effector and Cfluc398-Rho GTPase by site-directed mutagenesis. The catalytic domains of the regulator proteins (GEFs and GAPs) of Rho GTPases were amplified and cloned into pCMV-HA (Clontech). The related point mutations used in our experiment were introduced by PCR-mediated site-directed mutagenesis using the QuickChange kit. All of the Rho GTPases and the associated proteins (effectors, GEFs and GAPs) are human original. The graphic schemes of the various constructs are shown in Figure 1. The detail construction schemes and the plasmids of these BiLC-based Rho GTPase biosensors are available upon request. And the sequences of the important sensors have been submitted to the GenBank. The accession numbers of Cfluc398-CDC42_(F37A)_, Cfluc398-CDC42_(G12V)_, Cfluc398-CDC42_(T17N)_, Cfluc398-CDC42_(WT)_, Cfluc398-RacI_(F37A)_, Cfluc398-RacI_(G12V)_, Cfluc398-RacI_(T17N)_, Cfluc398-RacI_(WT)_, Cfluc398-RhoA_(F39A)_, Cfluc398-RhoA_(G14V)_, Cfluc398-RhoA_(T19N)_, Cfluc398-RhoA_(WT)_, Nfluc416-WASP, Nfluc416-PAK and Nfluc416-PKN are KC736072-KC736086.

### Cell Culture

Human embryonic kidney HEK 293 and mouse fibroblast NIH 3T3 cells were purchased from the American Type Culture Collection (ATCC; Manassas, VA, USA). All these cells were cultured in Dulbecco modified Eagle medium (DMEM, Gibco Laboratories, Grand Island, NY) supplemented with 10% fetal bovine serum (Gibco). Cell cultures were maintained in a 37°C incubator with 5% CO_2_.

### Cells-Based in Vitro Assay

Transfections were performed in 80% confluent 24-h-old cultures of HEK 293 or NIH 3T3 cells, which were plated on white, clear-bottom 96-well plates (Costar 3610; Corning, Inc., Corning, NY, USA). For transfection, 50 ng/well of biosensors (a pair of plasmid vectors) as indicated were transfected using Lipofectamine 2000 reagent according to the manufacturer's instructions. The renilla luciferase plasmid (pRL-tk, 2 ng/well) was cotransfected to normalize transfection efficiency. The cells were assayed after 24-h incubation at 37°C at 5% CO2. To image in living cells, after administration of D-luciferin (150 ug/ml in Cell Culture Medium, 100 ul/well), luminescent signal intensity (photons/second/square centimeter/steridian or p/s/cm^2^/sr) was measured by the charge-coupled device (CCD) camera of IVIS spectrum (Caliper Life Sciences, Hopkinton, MA) using the following parameters: 1-min exposure; emission filter, 600 nm; f-stop, 1; binning, 8; field of view, 15 cm. Renilla luciferase (RL) activity was measured by adding coelenterazine (1.5 uM in D-PBS, 100 ul/well) with the CCD camera (30 s exposure; emission filter, 500 nm; f-stop, 1; binning, 8; field of view, 15 cm). The measure of RL activity was preferential in order to avoid mutual interference, because RL emission signal intensity is almost negligible at 600 nm. Data for each well are expressed in the relative luminescence ratio, which is calculated as the ratio of the luminescent intensity of firefly luciferase (FL) at 600 nm to that of renilla luciferase (RL) at 500 nm (FL/RL). For the analysis of GEF and GAP activities in 96-well plates, 100 ng/well of expression vectors encoding these regulatory proteins or, as a control, the corresponding empty expression vector (pCMV-HA) were cotransfected with renilla luciferase vector and the biosensor pairs (Nfluc416-WASP/Cfluc398-CDC42_(WT)_, Nfluc416-PAK/Cfluc398-RacI_(WT)_ or Nfluc416-PKN/Cfluc398-RhoA_(WT)_). For the respond analysis of extracellular factors, NIH3T3 cells were plated on white, clear-bottom 96-well plates and transfected with 50 ng of the biosensor pairs (Nfluc416-WASP/Cfluc398-CDC42_(WT)_, Nfluc416-PAK/Cfluc398-RacI_(WT)_ or Nfluc416-PKN/Cfluc398-RhoA_(WT)_) per well. The cells were serum-starved (growth in serum-free DMEM medium for 6 h) and then detected the luminescence intensity. When the luminescence intensity became steady, we stimulated the cells with insulin (2 mg/ml), lysophosphatidic acid (LPA) (40 ng/ml) or bradykinin (100 ng/ml), which are the known activator of Rac1, RhoA and Cdc42, respectively [3], and then immediately acquired the sequence image using IVIS spectrum (1-min exposure; emission filter, open; f-stop, 1; binning, 8; field of view, 15 cm) for 30 min. In ligand titration experiments, the cells were stimulated with different concentrations of the stimulators and the luciferase signals were acquired after 3 min by IVIS spectrum (1-min exposure; emission filter, open; f-stop, 1; binning, 8; field of view, 15 cm).

### Western Blotting and coimmunoprecipitation

HEK 293 cells were plated on 100-mm culture dishes, grown to 80% confluence and then transfected with 2 ug of BiLC biosensors. After incubation for 24 h, the cells were harvested in cell lysis buffer (20 mM Tris, pH 7.5, 150 mM NaCl, 1% Triton X-100, 2.5 mM sodium pyrophosphate,β-glycerophosphate, 1 mM EDTA, 1 mM Na_3_VO_4_, 1 ug/ml leupeptin, 1 mM phenylmethylsulfonyl fluoride). One part of the whole-cell lysates was directly subjected to SDS-PAGE and probed with a 1∶1000 dilution of goat anti-luciferase polyclonal antibody (anti-luciferase pAb, Promega, catalog #G745A) to confirm the expression of the biosensors. The primary antibody was detected with a 1∶2000 dilution of HRP-conjugated donkey anti-goat IgG (Promega, catalog #V805A). Blots were developed using enhanced chemiluminescence (ECL) reagent (Amersham BioSciences). The proteins in the remaining lysates were coimmunoprecipitated with mouse anti-myc antibody (clone 4A6; Millipore). The immune complexes were captured using protein G-coupled magnetic beads (Millipore) and then fractionated by SDS-PAGE. Nfluc-effectors and Cfluc-Rho GTPases were detected with anti-myc and anti-luciferase polyclonal antibody, respectively.

### In Vivo mouse imaging experiments using pseudotumors

All the mice used were 6-week-old. 293 cells plated in 100-mm dishes were transiently cotransfected with pRL-tk vector and BiLC biosensors as indicated and implanted subcutaneously 24 h later. The cell numbers (about 1×10^7^) of implantation were confirmed by luminescent intensity of RL activity in vitro, making sure the relative consistency among different groups. After a residence time of 24 hours, D-luciferin was injected intraperitoneally at 150 mg/kg BW. The mice were imaged using IVIS spectrum (3-min exposure; emission filter, open; f-stop, 1; binning, 8; field of view, 15 cm).

## Results and Discussions

### Overview of the bioluminescent Rho GTPase biosensors based on spilt luciferase complementation

Because of their important and complicated functions in physiological and pathophysiological processes, the imaging of GTPases is becoming a new research hotspot, since Mochizuki, N., et al. introduced a method to image the activation of Ras and Rap1 [24]. In the last decade, the designs of GTPase biosensors based on fluorescent imaging contain two kinds: the “GTPase-effector fusion” design and the “effector domain only” design [7], [25]. The former design requires ectopic expression of the labeled GTPases, and does not directly reveal the endogenous GTPases [7]. But the exogenous GTPase can mimic the function of the endogenous GTPase and respond to the upstream signals through GEFs or (and) GAPs (Figure 1C). And we can discriminate whether the special GTPase is involved in this signaling pathway or not, just because of the specificity of the particular GTPase-effector partner. Therefor, this design is widely used to develop FRET Rho GTPase sensors [26]. In contrast, the “effector domain only” sensor suffers from the lack of specificity inherent to the design [7]. Many effectors have multiple specificities, such as WASP can interact with CDC42 and CT10; PAK can interact with CDC42 and RacI; and PKN can interact with RhoA, RhoB and RhoC. It's difficult to discriminate which GTPase induced the signal change. But, in theory, this sensor can measure the activity of endogenous GTPase, though the results were not as satisfied as expected [7], [9].

The split luciferase fragment-assisted complementation technique is thought to have the most sensitive and highest dynamic range due to the enzymatic amplification of signals and the optimized bio-compatible substrate (high cell permeability and high quantum yield) among all the protein-protein interaction (PPI) detection methods [27], [28], and has been successfully used to investigate many signal transduction pathways in mammalian living cells [29], [30], [31]. And it is just suitable for the “GTPase-effector fusion” design. Hence, this technique was employed by us to visualize Rho GTPase pathways, which are closely relative to oncogenic transformation, invasion, tumorigenesis and other diseases. The strategy is to reasonably split luciferase into two non-functional fragments and fuse these two fragments, in fame and with a short flexible linker (G_2_S)_2∼4_ or (G_4_S)_1∼2_ [22], to Rho GTPase and the GBD of the specific effector, respectively. Once Rho GTPase is activated by upstream stimulating factors and subsequently binds to the GBD of the effector, the two luciferase fragments are brought into close proximity, leading to luciferase reconstitution and photon production in the presence of the substrate (Figure 1C). And the activity change of Rho GTPases can be monitored and quantified by indirectly estimating the reconstituted bioluminescence activity. In order to demonstrate that GTP-loading on Rho can increase the luminescent intensity, a series of biosensors carrying either the wild-type or various mutants of Rho GTPases were prepared in this experiment. In the proteins denoted with the suffix G12V or G14V, Gly^12^ of CDC42 and Rac1 or Gly^14^ of RhoA was replaced with Val to inactivate the GTPase activity, resulting in constitutively activated forms (dominant positive mutants). In T17N or T19N mutants, Thr^17^ of CDC42 and Rac1 or Thr^19^ of RhoA was replaced with Asn. These mutations are known to reduce the affinity of G proteins for guanine nucleotides and downregulate Rho GTPase activity (dominant negative mutants) [32]. In F37A or F39A mutants, Ala was substituted for Phe^37^ or Phe^39^ in the effector domain of Rac and Cdc42 or RhoA, which is essential for the binding to the specific effector (effector-loop mutants) [33]. Although initially firefly luciferase and renilla luciferase were tested as prospective bioluminescent reporters, renilla luciferase was eventually discarded due to its low reconstituted luminescence activity. We chose the split renilla luciferase fragments (Nrluc229/Crluc229) [34] to detect the interaction of CDC42 and WASP, but we didn't observed the anticipated luciferase complementation in living cells (data not shown). On the other hand, firefly luciferase is more suitable for application in studying signal transduction pathway of tumor cells than renilla luciferase, because the latter emits blue light, which is highly attenuated in living tissue, and its' substrate, coelenterazine, has been shown to be transported by the multi-drug resistance transporter P-glycoprotein [35], which are widely expressed in tumor cells.

### Optimizing the configuration of BiLC-based biosensors for visualizing Rho GTPase signaling pathway

**Figure 2:**
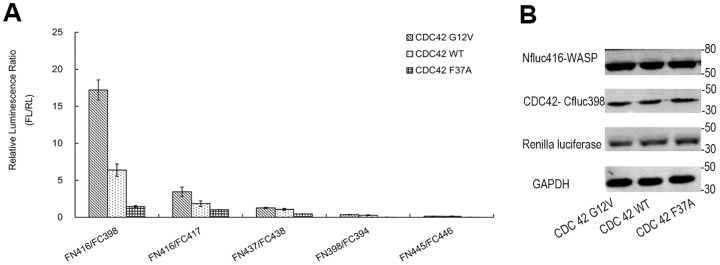
Optimizing appropriate dissection site of firefly luciferase for BiLC Rho GTPase biosensor. (A) The optical imaging results among different split-sites of firefly luciferase. Relative luciferase activities were detected in living 293 cells cotransfected with the five different combinations of split firefly luciferase fragments (Nfluc416-WASP/Cfluc398-CDC42, Nfluc416-WASP/Cfluc417-CDC42, Nfluc437-WASP/Cfluc438-CDC42, Nfluc398-WASP/Cfluc384-CDC42, Nfluc445-WASP/Cfluc446-CDC42), respectively. In each luciferase fragments combination, luciferase activity was compared among CDC42 WT, G12V, and F37A mutants. WT, G12V, and F37A indicate wild type, the constitutively active mutant, and the effector-loop mutant, respectively. The results were normalized using cotransfection of renilla luciferase plasmid (pRL-tk) and represented by the ratio of luminescent intensity of firefly luciferase (FL) at 600 nm to that of renilla luciferase (RL) at 500 nm (FL/RL). The data shown are representative of four separate experiments performed with quadruplicate culture wells. The result shows that the combination (Nfluc416/Cfluc398) had the widest dynamic range and the highest luciferase activity restoration. (B) The western blots carried out in parallel to demonstrate the protein expression among CDC42 WT, G12V, and F37A biosensors. This figure only shows the results of Nfluc416-WASP/CDC42-Cfluc398 as representative.

To demonstrate the feasibility of BiLC-based biosensors for imaging Rho GTPase signaling pathways, a pair of known interacting proteins (CDC42 and the GBD (AA 220–288) of its effector WASP) was selected beforehand to put this strategy into practice, attempting to explore and construct the optimal configuration. The three dimensional structure (PDB ID: 1EJ5) of WASP GBD bound to Cdc42 indicates that if the amino terminal fragment of firefly luciferase (FN) was fused upstream of WASP-GBD and the carboxyl terminal fragment (FC) was added downstream of CDC42, the two luciferase fragments could be sufficiently brought together by the interaction of CDC42 and WASP and then yield significant complemented luciferase enzyme signal. We speculated that this configuration (Nfluc-WASP/CDC42-Cfluc) may be a good domain arrangement. As with other protein complementation assay methods, one of the key prerequisites for the split luciferase complementation strategy is an appropriate dissection site [36]. Based on this predicted domain arrangement, we first tested five prospective dissection sites, which have been reported by others [22], [37], [38], [39]. The graphic schemes of these various constructs are presented in Figure 1A. The result shows that the split firefly luciferase fragments (Nfluc416/Cfluc398), which has the widest dynamic range and the highest luminescent signal, is suitable to construct CDC42 biosensors based on BiLC strategy (Figure 2). This result also demonstrates the overlapping fragments are very critical to reconstitute luciferase activity for BiLC-based biosensors. It's worth mentioning that when we transfected cells with individual fusion construct (Nfluc416-WASP or CDC42-Cfluc398) as well as unfused Nfluc416/Cfluc398 pair, no detectable bioluminescence relative to untransfected cells was detected by the CCD camera of IVIS spectrum (data not shown), indicating the background bioluminescence was indeed very low [22].

**Figure 3:**
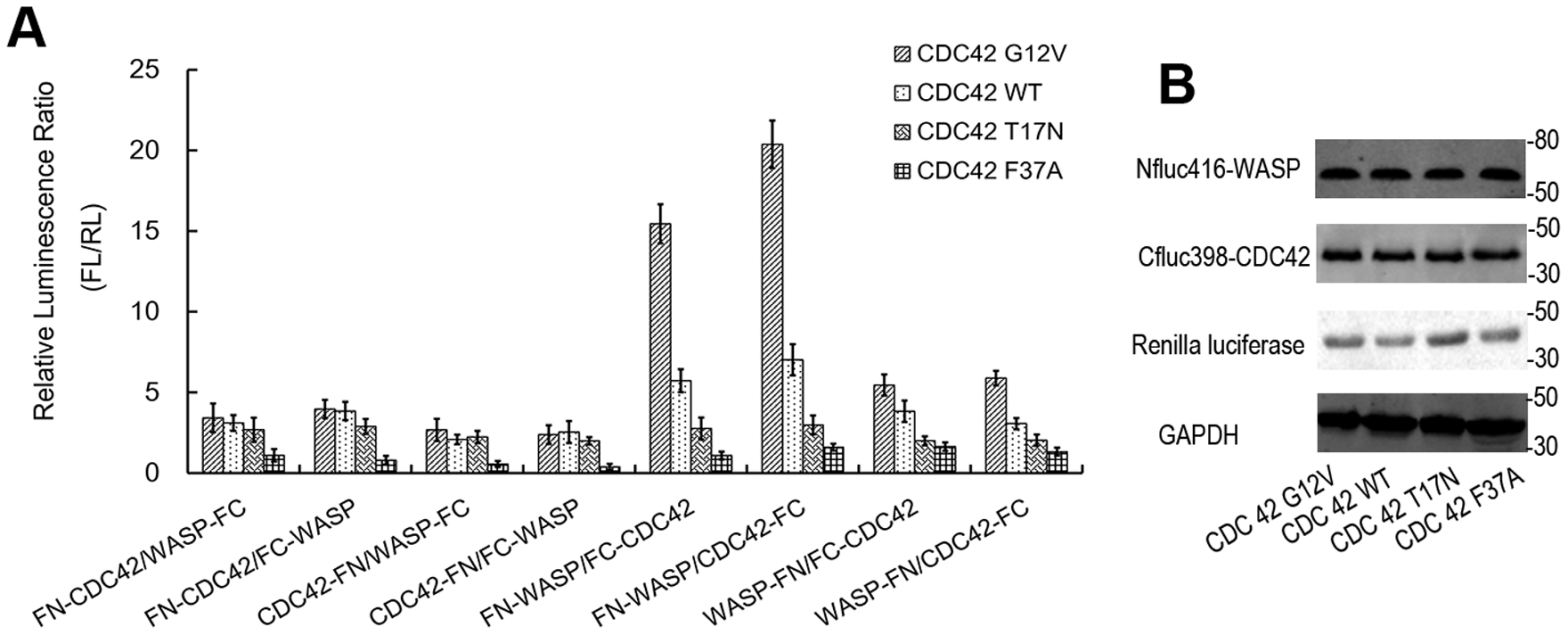
Optimizing the appropriate configuration (or domain arrangement) for BiLC Rho GTPase biosensor. (A) The optical results of eight different configurations. Relative luciferase activities were detected in living 293 cells cotransfected with the different configurations of N- and C-terminal FL fragments with interacting proteins CDC42 and WASP constructed with different orientations. Correct configuration is very critical to achieving efficient reconstruction of luciferase activity with PCA strategy. In each configuration, luciferase activity was compared among CDC42 WT, G12V, T17N and F37A mutants, which represent different levels of CDC42 activity and different degrees of the interaction. WT, G12V, T17N and F37A indicate wild type, constitutively active mutant, dominant negative mutant and effector mutant, respectively. The results were normalized using cotransfection of RL and represented by the ratio of luminescent intensity of FL at 600 nm to that of RL at 500 nm. The data shown are representative of three separate experiments performed with quadruplicate culture wells. The results show that the combinations containing Nfluc416-WASP/CDC42-Cfluc398 and Nfluc416-WASP/Cfluc398-CDC42 produced a greater level of luminescent signal and wider dynamic rang for different levels of CDC42 activity. (B) The western blots carried out in parallel to demonstrate the protein expression among CDC42 WT, G12V, T17N and F37A biosensors. This figure only shows the results of Nfluc416-WASP/Cfluc398-CDC42 as representative.

To acquire more efficient complementation-assisted luciferase enzyme activity using this BiLC strategy, another problem that should be considered is that which end (amino or carboxy terminal) of the interacting proteins (such as CDC42 and WASP) need to be fused to which luciferase fragments (FN or FC) [40]. The appropriate orientation of FN and FC reporter fragments and the interacting proteins is very critical to achieving efficient complementation [41]. Although we have obtained the good results through the speculative configuration (Nfluc-WASP/CDC42-Cfluc), we further strived to search for the optimal domain arrangement of the split FL fragments and the interacting protein pair. Because this configuration has a hidden problem that it's not sensitivity to RhoGDI activity, like “Raichu” biosensors [7]. It's not only the carboxy terminal CAAX motif but also the switch domains (switch I and switch II) of Rho GTPase are required for RhoGDIs to regulate the GDP/GTP cycle and the membrane association/dissociation cycle [42] . Hence, to search for the configuration most suitable for the Rho GTPase biosensors, we constructed biosensors with all possible configurations based on the preferred dissection site (Nfluc416/Cfluc398) (Figure 1B). All these biosensors were studied in 293 cells by transient cotransfection. In each configuration, we compared the reconstituted bioluminescence of the four different alleles of CDC42, which represent different activity states of CDC42 and different interaction degrees between CDC42 and WASP. Figure 3 shows that the configuration Nfluc416-WASP/CDC42-Cfluc398 had the strongest luciferase activity and the widest dynamic rang (CDC42 G12V is 2.9-fold greater than CDC42 WT, 6.8-fold greater than CDC42 T17N and 12.7-fold greater than CDC42 F37A). And the configuration Nfluc416-WASP/Cfluc398- CDC42 also generated a wider dynamic range among the four different forms of CDC42 (CDC42 G12V is 2.7-fold greater than CDC42 WT, 5.6-fold greater than CDC42 T17N and 14. 3-fold greater than CDC42 F37A). Generally, it's difficult to reach such a big dynamic rang for FRET assay, so these BiLC-based biosensors may be more advantageous to investigate Rho GTPase signaling pathways than the intramolecular FRET systems reported by others [9], [12]. Therefore, we boldly speculated that the configurations of Nfluc416-effector/Rho GTPase-Cfluc398 and Nfluc 416-effector/Cfluc398-Rho GTPase may be the preferred mode designs for constructing Rho GTPase biosensors based on BiLC strategy. However, to circumvent the weakness of GDIs' regulation aforementioned, we eventually employed the configuration of Nfluc 416-effector/Cfluc398-Rho GTPase as the most optimal configuration to construct BiLC-based Rho GTPase biosensors.

### Application of BiLC-based biosensor to imaging other different Rho GTPases

**Figure 4:**
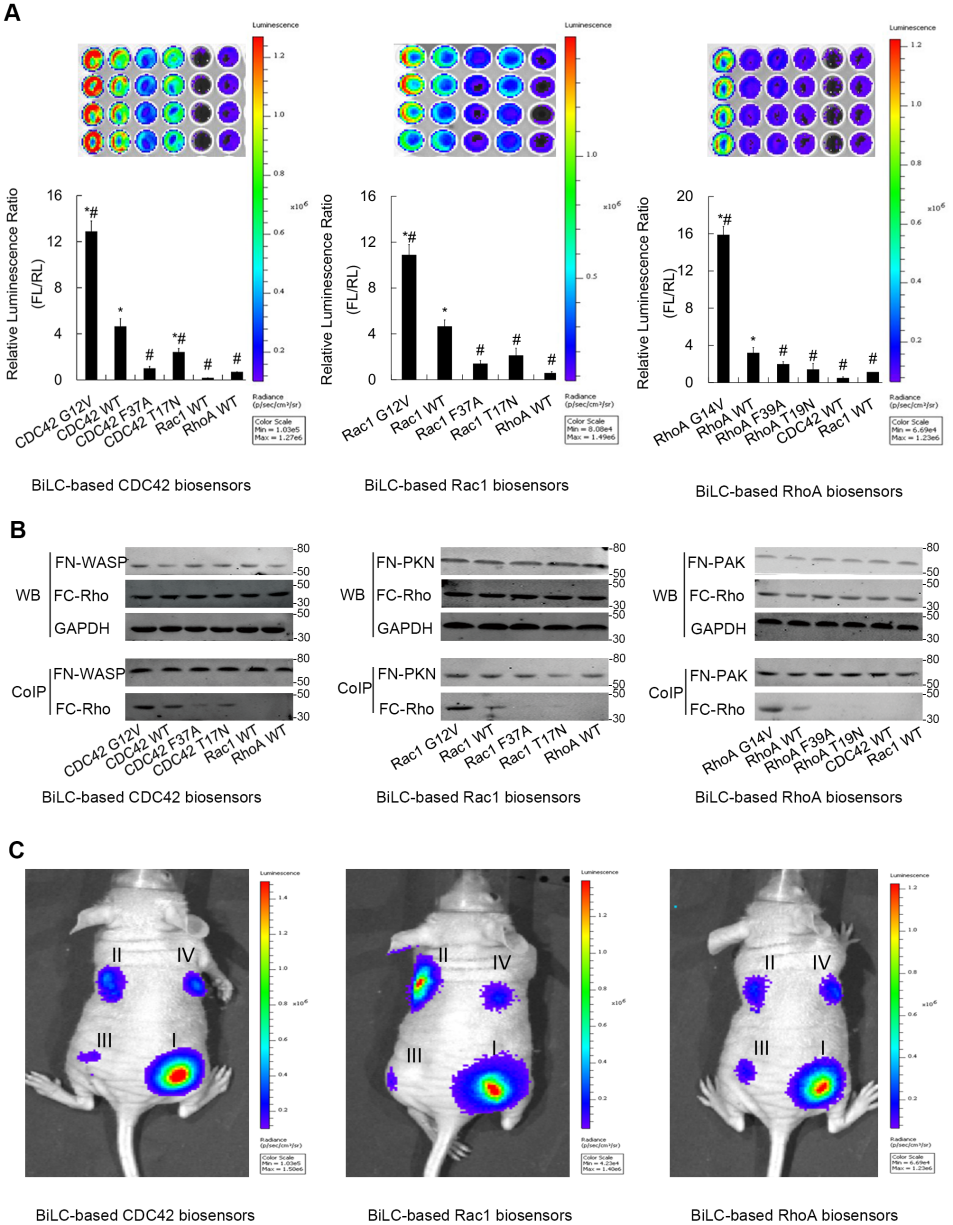
The application of BiLC strategy to image the three main members of Rho GTPases. (A) The results of optical imaging of three kinds of BiLC RhoGTPase biosensors. The relative luminescence was calculated by the ratio of luminescent intensity of firefly luciferase (FL) at 600 nm to that of renilla luciferase (RL) at 500 nm (n = 4, representative of 4 independent experiments). Error bars denote standard deviations. Asterisks (*) denotes samples that show a difference from the nonspecific complementation (the non-interactive GTPase-effector pairs or the effector loop mutants) with statistical significance by analysis of variance (ANOVA) (*p*≤0.01). This result indicates that the nonspecific complementation does not impede the correct interpretation of effective interactions induced by GTPase activation. WYJH (#) denotes samples that show a difference from the wild-type biosensor with statistical significance by ANOVA (*p*≤0.01). This result indicates that the BiLC sensors possess the discriminatory power among different GTPase activity states. (B) The results of coimmunoprecipitation. The results show that the expressions of the BiLC biosensors had no significant discrimination among different alleles of Rho biosensor, but the intensities of the PPIs displayed obvious diversities, which were in accordance with the results obtained by our optical imaging. (C) In vivo optical CCD imaging of BiLC Rho GTPase biosensors. The pseudotumors in living mice were generated by engrafting with transiently transfected 293 cells. The pRL-tk plasmid was cotransfected and RL activity was detected to normalize the planted cell number. 24 h after implantation, the mice were imaged using IVIS spectrum. A significant discrimination of luciferase activity was detected among different alleles of Rho GTPase. (I: the dominant active mutants; II: the wild-type Rho GTPases; III: the effector-loop mutants; IV: the dominant negative mutants).

To demonstrate the universality of BiLC strategy in visualizing Rho GTPases pathways, we further used this optimal configuration (Nfluc416-effector/Cfluc398-RhoGTPase) to construct Rac1 biosensor (Nfluc416-PAK/Cfluc398-Rac1 and mutants thereof) and RhoA biosensor (Nfluc416-PKN/Cfluc398-RhoA and mutants thereof). Therefor, we obtained a Rho GTPase biosensor system which can image the three best characterized Rho GTPases (CDC42, Rac1 and RhoA). All these sensors were studied in 293 cells by transient cotransfection under appropriate conditions. Firefly luciferase activities were imaged using the CCD camera of IVIS spectrum, and the pseudo-color images representing light intensities (blue: least intense; red: most intense) were generated by the Living Image® 4.2 software program (Caliper Life Sciences, Hopkinton, MA). As is shown in Figure 4A, a significant activity restoration appears in three kinds of Rho GTPase biosensor. As a control, will-known non-interactive GTPase-effector pairs (such as RhoA/WASP, RhoA/PAK, CDC42/PKN, RacI/PKN and RacI/WASP) were introduced to characterize the background bioluminescence of nonspecific complementation, which was caused by the high concentration of the biosensors in local region [29]. Nevertheless, in our experiment, this background bioluminescence is significantly lower than that of the effective interactions (wild-type and/or constitutively active mutant). This result indicates that the nonspecific complementation does not cloud the correct interpretation of the effective interaction induced by Rho GTPase activation. And more importantly, there was a large dynamic range among different allele of GTPase, which represent the different activity states of GTPases. Especially, the luciferase activities yielded by the constitutively active mutants (G12V or G14V) were significantly higher than that of the wild-types, the dominant-negative mutants (T17N or T19N) and the effector-loop mutants (F37A or F39A). This result indicates that the BiLC-based biosensors possess the discriminatory power among different GTPase activity states. It's worth noting that the luciferase activities of the dominant-negative mutants were also obvious, especially for CDC42 and Rac1 biosensors. But this phenomenon was not seen among the effector-loop mutants (F37A or F39A). This may be due to the high sensitivity of the BiLC biosensors and the different mechanism of inactivation. The dominant negative mutants (T17N or T19N) only reduce the affinity of GTPase to GTP[32] and preferentially bind GDP rather than GTP[43]. Although they are thought to exist constitutively in the GDP-bounds (inactive), there are more or less GTP-bounds (active) remaining. Because of the enzymatic amplification of signals inherent to BiLC, the a few remaining activity was magnified. In contrast, the effector-loop mutants are substituted the critical amino-acid in Switch I and directly abolish the ability to interact with downstream effector [33]. And we can also detect the luciferase activities by wild-type biosensors. This is due to the basal activation of Rho GTPase in the physiological environment. To further strengthen our results, western blotting was used to confirm the expression of the biosensors, and coimmunoprecipitation was performed to assess the interaction information between the two portions of the BiLC-based biosensors. As is shown in Figure 4B, the expressions of the BiLC biosensors had no significant discrimination among different combinations in our transfection experiments. Accordingly, the amount of Cfluc-Rho GTPase coimmunoprecipitated with Nfluc416-effector displayed diversity, in accordance with the luminescent intensity obtained by our optical imaging. On the other hand, the overwhelming preponderance of bioluminescence compared to fluorescence is the sensitiveness of in vivo imaging because of no autofluorescence. Hence, we checked check the feasibility of its optical discrimination for different levels of Rho GTPase activity in living animals. We implanted the nude mice with 293 cells transfected with BiLC biosensors, constructed pseudotumors and acquired luciferase signal by IVIS spectrum. As is shown in Figure 4C, a significant discrimination of luciferase activity was detected in vivo among different alleles of Rho GTPase, which represent the different GTPase activity. This indicates that the BiLC-based Rho GTPase biosensors have great potential for application in vivo.

Unlike FRET, photon is produced by luciferase oxygenating the substrate in BiLC. Because no excitation is used, there is no autofluorescent background, no bleaching of the donor fluorophores and no co-excitation of the acceptor fluorophores by the energy used to excite the donor [44]. And, as a protein complementation assay (PCA), BiLC have the ‘on or off’ nature of signal, which is unlike the gain of FRET. These features offer BiLC a high signal-to-noise ratio [45]. In addition, the enzymatic reaction of luciferase may amplify the minor differences of the signals. Thus, the merits make the BiLC biosensors as a highly sensitive method for imaging Rho GTPase pathways than FRET probes. In our experiments, the dynamic ranges of the signal values between the active mutants and the wild-types are respectively 2.77-fold, 2.34-fold and 5.15-fold for CDC42, RacI and RhoA. Without regard to the experimental conditions and the cell lines, these results may be higher than that of the FRET probes (about 1.4-fold, 1.3-fold and 1.6-fold for CDC42, RacI and RhoA, respectively) [12], [23]. The low sensitivity of FRET primarily because the gain of FRET probes does not generally exceed 50% [18], [46].

But just as a coin has two sides, BiLC probes usually require several minutes to respond to the stimulus, and an even longer time to return to the baseline level. In contrast, FRET provide a high temporal resolution for Rho GTPase activity in live cells on the order of single seconds[47]. And the BiLC biosensors based on luciferase is also not good at revealing subcellular location, although Kaihara A. et al. used the split renilla luciferase complementation method to located the interaction between Y491 and SH2n near the plasma membrane using a cooled charge-coupled device camera [48]. The high temporal resolution and spatial resolution enable FRET Rho GTPase probes very suitable for the detection of activities in protrusive areas, such as lamellipodia and filopodia [7], [47]. However, the a broader application of these probes may be limited by some disadvantages, such as autofluorescence and challenges of stable expression [18], especially for in vivo imaging and high-throughput screening. Because of high homology, the DNA of CFP and YFP frequently recombine during the integration to the genome [18]. Without this recombination phenomenon, BiLC sensors can easily be stably expressed and applied to in vivo imaging. Moreover, it will be possible to visualize the cross-talk between Rho GTPases using the multicolor luciferase complementation [49], [50]. As a last resort, luciferase complementation assays also can provide general applicability in protein interactions with considerable spatial and temporal resolution in opaque or strongly autofluorescent living subjects [51]. Therefor, as a novel alternative, the BiLC Rho GTPase sensor is a good supplement to the FRET probe.

### Analysis of the responses of BiLC-based Rho GTPase biosensors to upstream regulatory proteins and extracellular ligands

In the BiLC sensor, the fragment of luciferase is fused to the Rho GTPase. This can affect the interaction of GTPase with downstream effectors and upstream regulatory proteins (such as GEFs and GAPs). By trial and error, we obtained the optimal configuration (Nfluc416-GBD/Cfluc398-WASP) and successfully apply this configuration to develop a system which can image the three best characterized mammalian Rho GTPases. The restoration of luciferase activity and the coimmunoprecipitation (see Figure 4) have demonstrated that the Cfluc-RhoGTPase fusion proteins can mimic the endogenous GTPases and interact with the binding domains of downstream effectors. It's worth noting that this non-physiological interaction more or less interferes with the downstream function [18]. This interference won't completely eliminate in molecular imaging, but appropriately controlling the expression of molecular probe is necessary to minimize the flaw.

**Figure 5:**
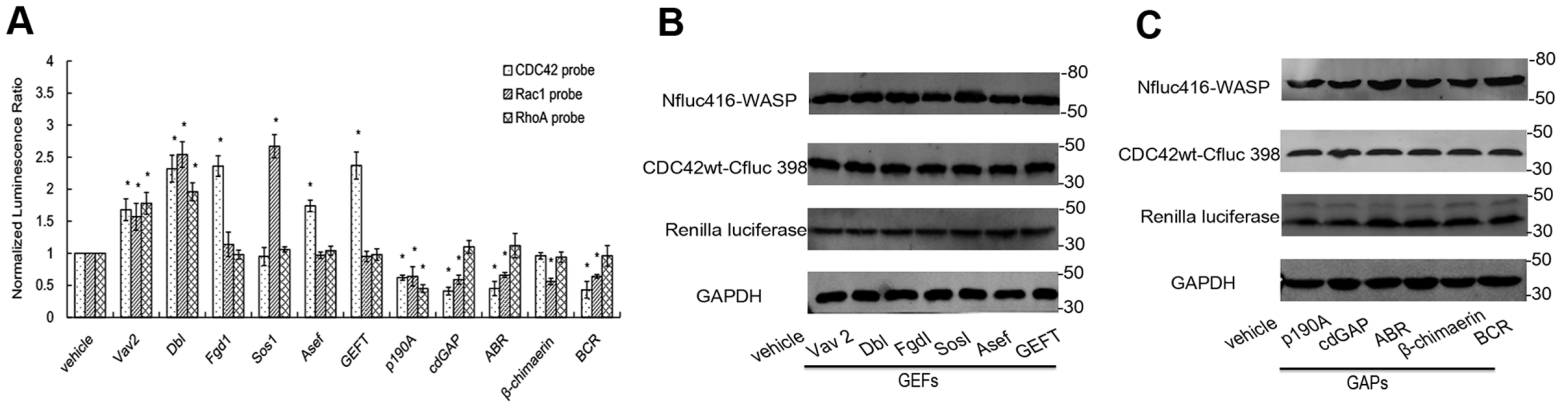
The sensitivity analysis of BiLC Rho GTPase biosensors to GEFs and GAPs. (A) The results of optical imaging among different upstream regulatory proteins. The luminescent signals were normalized using cotransfection of renilla luciferase plasmid (pRL-tk) and represented by the ratio of luminescent intensity of firefly luciferase (FL) at 600 nm to that of renilla luciferase (RL) at 500 nm. The final results were normalized by the luminescence ratio of the control vehicles, which were designated with “1”. Data is reported as the fold increase in luminescence ratio (FL/RL) relative to control. Error bars denote standard deviations. Asterisks (*) denotes samples that show a difference from the control vector with statistical significance by analysis of variance (ANONA) (*p*≤0.01). The data shown was obtained by three separate experiments performed with quadruplicate culture wells. The results highly accord with the well-known experimental data, indicating that the BiLC biosensors can response to the upstream regulatory molecules. And this ability of BiLC GTPase sensors can be used to examine the substrate selectivity of GEFs and GAPs and quantify their catalytic activities in intact living cells. (B and C) The western blots carried out in parallel to demonstrate protein expression among different GEFs and GAPs. The figure only shows the results of the CDC42 biosensors as a representative.

In the aforementioned studies, we only used different mutants to manipulate the activity states of Rho GTPases and demonstrated the restoration of luciferase enzyme activity in a GTP-dependent manner. This doesn't necessarily justify the Cfluc-RhoGTPase fusion proteins can correctly interact with upstream regulatory proteins. To test the rigorous validity of our BiLC-based biosensors, we further tested the responses of these biosensors to upstream regulatory proteins (GEFs and GAPs) and extracellular ligands in living cells. Because BiLC strategy is generally performed as plasmid-based transfections, it works well in conjunction with other target genes requiring transfection, such as plasmid encoding regulatory proteins of GTPases. Thus, in this section, we experimentally tested the responses of this biosensor system to GEFs and GAPs, to justify its sensitivity to the upstream signal transduction. The catalytic domains of six well-known GEFs (Dbl, Fgd I, Asef, GEFT, Sos1 and Vav1) and five GAPs (p190A, cdGAP, ABR, β-chimaerin and BCR) were used as the representatives. Through these molecules, we can objectively test the sensitivity of this system to monitor GEF-catalyzed nucleotide exchange and GAP-stimulated GTP hydrolysis in living cells. Data are expressed as a ratio of experimental to control values, in order to facilitate comparison between activities from different regulator molecules. Figure 5 illustrates the luciferase activity of BiLC CDC42 biosensors significantly increased in the presence of Vav2, Dbl, Fgd1, Asef and GEFT, demonstrating the GEF activities of these proteins toward CDC42. In addition, our assays also indicated that Dbl, Vav2 and Sos1 could activate Rac1; and Dbl, Vav2 worked on RhoA. Accordingly, for the GAP activities of the five previously characterized GAPs, our observations is that p190A catalyzed the GTP hydrolysis of all three GTPases, cdGAP, ABR and BCR regulated both CDC42 and Rac1, β-chimaerin specifically stimulated the GTPase activity of Rac1. These results highly accord with the well-known experimental data. Thus, the BiLC biosensors can mimic the function of endogenous GTPases to receiving upstream signal and correctly response to the stimulation. Meanwhile, we can exploit this feature to identify the substrate selectivity of a new GEF or GAP and quantify their catalytic activities in living cells just like ‘pull-down’, but in a fast and simple way.

**Figure 6:**
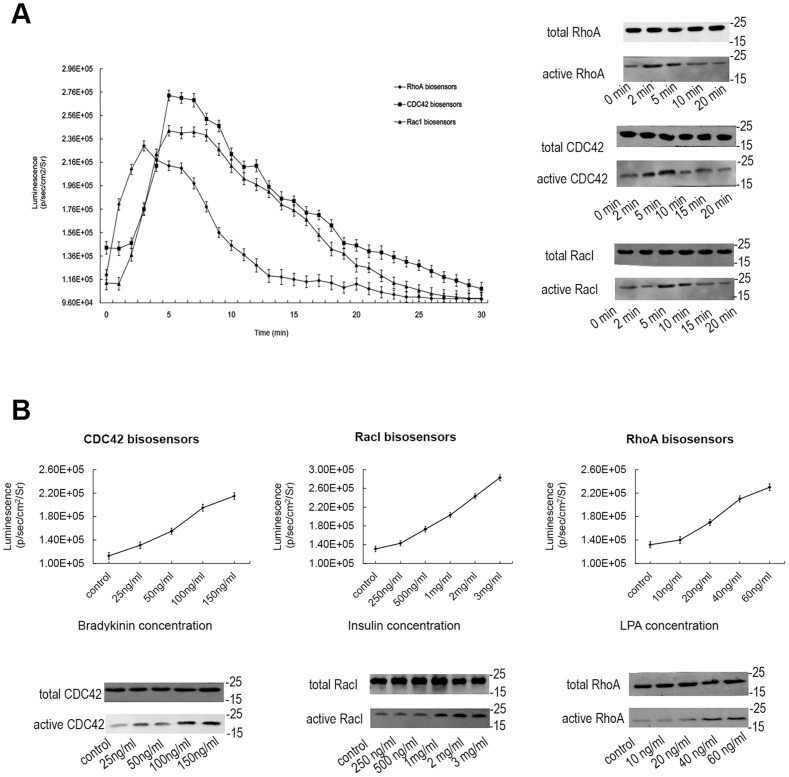
The sensitivity analysis of BiLC Rho GTPase sensors to extracellular ligands. (A) The temporal response of BiLC Rho GTPase sensors stimulated by extracellular ligands. After being serum-starved in serum-free DMEM medium for 6 h, mouse fibroblast NIH3T3 cells were detected the luminescent signals by adding D-luciferin until the intensities became steady, then stimulated with insulin (2 mg/mL), lysophosphatidic acid (40 ng/mL) and bradykinin (100 ng/mL), which are the known activator of Rac1, RhoA and Cdc42 respectively, and then immediately acquired the sequence image (1-min exposure; emission filter, open; f-stop, 1; binning, 8; field of view, 15 cm) for 30 min using IVIS spectrum. The data shown were obtained by three separate experiments performed with quadruplicate culture wells. The result shows that not only the activation signals of Rho GTPases from upstream pathways but also the subsequent decrease following the hydrolysis of GTP can be displayed and quantified by the BiLC-based biosensors. And the optical results (left) accord with that of ‘pull-down’ in our previous work (right). (B) The responses of BiLC Rho GTPase sensors to different concentration of extracellular ligands. The cells were stimulated with different concentrations of the stimulators and the luciferase activity was acquired after 3 min by IVIS spectrum (1-min exposure; emission filter, open; f-stop, 1; binning, 8; field of view, 15 cm). The data shown was obtained by three separate experiments performed with quadruplicate culture wells. The optical results (upper) were in accordance with that of our previous ‘pull-down’ (under).

To further validate whether the system is sensitive to the upstream signals, we tested its responses to the stimulations of extracellular ligands. In this experiment, we use insulin, lysophosphatidic acid and bradykinin as the extracellular stimulators, which are the known activator of RacI, RhoA and CDC42, respectively [32]. And the stimulant concentrations were obtained by pull-down in our previous works (Figure 6B). Luminescences were quantified by drawing regions of interest and measuring light emission as p/s/cm^2^/sr. First, we proved that without stimulation, there was no obvious increase of luciferase activity restoration after luminescences became steady, and this steady signal began to gradually decrease after about 20∼30 min (data not shown). However, when we administrated the stimulators, the feature of response is completely different. As is shown in Figure 6A, a non-sustainable increase peak appeared for each kind of Rho GTPase sensor. However, the trends for the three kinds of Rho GTPase biosensors were not exactly same. In detail, for RhoA biosensors stimulating with lysophosphatidic acid, the luminescent signal increased rapidly to its peak after 3 min stimulation, and then soon returned to its initial level in 13 min. The CDC42 biosensors and Rac1 biosensors didn't respond as rapidly as the RhoA biosensors, and peaked after stimulation for 5 min, then gradually decreased to initial value in about 20 min. The results shows that the activation signals of Rho GTPases from upstream pathways can be indicated by our BiLC-based biosensors and displayed by the changes of luciferase activities in living cells. The reaction tends of the induced activations obtained by our BiLC biosensors well accord with the amounts of activated (GTP-bound) Rho GTPases examined by the pull-down method, although the luciferase activity seems to need longer time to return the baseline level. This may due to the inferior temporal resolution aforementioned. In addition, we used different concentrations of the stimulators to stimulate the BiLC biosensors after 3 min. As is shown in Figure 6B, the results were in accordance with our previous ‘pull-down’.

The experiments above completely demonstrated that the BiLC biosensor systems can correctly response to the upstream signaling, and the sensitivity is very high. Thus, we can easily reveal or quantify the signal change in the Rho GTPase pathways, by estimating the changes of luminescent intensity, without lysing cells and doing labor-intensive works inherent to ‘pull-down’. Although this system can't entirely displace the function of the tradition method, it saves a great deal of time and effort as a screening method. And unlike the bimolecular fluorescence complementation (BiFC) biosensors, BiLC biosensors do not assemble irreversibly [52]. Thus, our BiLC biosensors can detect not only the increase of Rho GTPase activity by stimulant treatment but also the subsequent decrease following the hydrolysis of the GTP-bound Rho GTPase in living cells. Because of these merits, the application of these biosensors to drug development would facilitate the high-throughput screening or identification of small molecules inhibitors or activators that targeted Rho GTPases or their upstream regulators.

## Conclusions

In this study, a new type of biosensor system for Rho GTPases based on bimolecular luminescence complementation has been established by optimizing the split site and the configuration. These genetically encoded biosensors are induced by Rho GTPase activation in a GTP-dependent manner. It's robustness has been demonstrated through visualizing the three best characterized Rho GTPases (CDC42, Rac1 and RhoA) in living cell and in vivo. It has also been proved that this system is sensitive to the upstream regulatory proteins (GEFs and GAPs) and the extracellular ligands. As a novel alternative method to investigate Rho GTPase-associated signaling pathways, it takes a lot less effort than traditional ways, such as “pull-down” or two-hybrid analysis. And, more importantly, due to its high sensitivity and no autofluorescent background, it can be directly applied to living mammalian cells and in vivo. These merits make it as a good supplement to traditional technologies and fluorescent imaging. Application of these BiLC-based biosensors or combination with other methods will facilitate our research works: for example, exploring how Rho GTPase signaling pathways operate in living cells and deciphering the complex function of Rho GTPase signal transduction in living animal (such as in tumor model). Meanwhile, by virtue of the properties and advantages of bioluminescence imaging, this system makes it possible to carry out high-throughput screening assay for identifying therapeutic agents targeted to Rho GTPase pathways. We also hope that the BiLC biosensors generated here may provide principles and basic methods for proper application of BiLC strategy to shed light on other small G proteins.
