# Expression of Concern: Novel Split-Luciferase-Based Genetically Encoded Biosensors for Noninvasive Visualization of Rho GTPases

**DOI:** 10.1371/journal.pone.0287871

**Published:** 2023-06-23

**Authors:** The PLOS ONE Editors

Following the publication of this article [1], concerns were raised regarding results presented in Figs 4 and 6. Specifically,

- The following Fig 4B panels appear similar: CDC42 biosensors WB FC-Rho, CDC42 biosensors CoIP FN-WASP when rotated 180°, and RhoA biosensors CoIP FN-PAK when flipped horizontally
- The following Fig 4B panels appear to partially overlap with one another: Rac1 biosensors WB FC-Rho, RhoA biosensors WB FC-Rho, and RhoA biosensors WB GAPDH when rotated 180°
- The following Fig 6A panels appear similar: Active RhoA, active Rac1, and active CDC42 when rotated 180° and vertically compressed

The corresponding author commented that errors were made during figure preparation, due to the similarities in bands and overall underlying images. The corresponding author also clarified that during their post-publication review of the data, they noticed that the following published panels were also incorrect:

- Fig 4B CDC42 biosensors WB FN-WASP
- Fig 4B Rac1 biosensors CoIP FN-PKN
- Fig 4B RhoA biosensors WB FN-PAK

**Fig 4:**
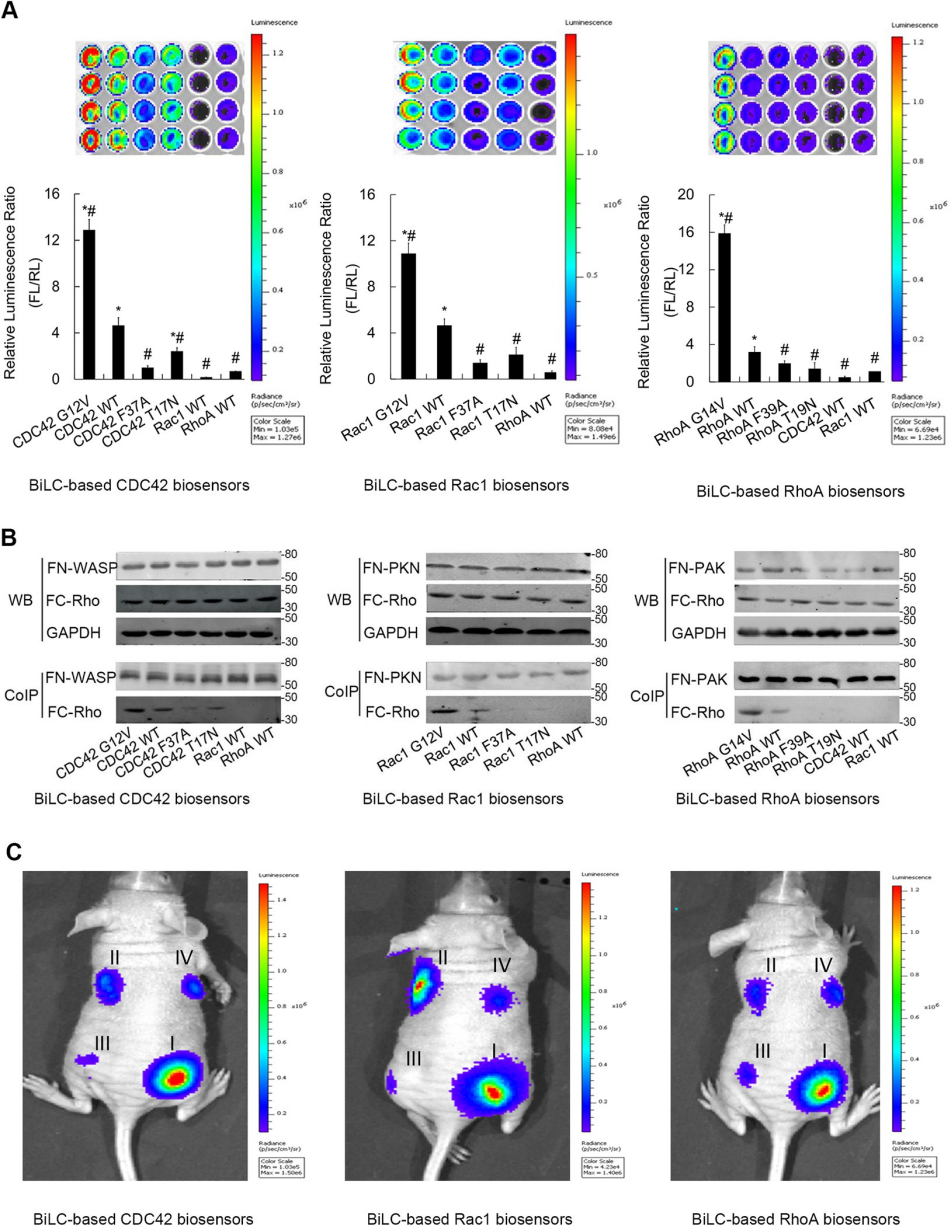
The application of BiLC strategy to image the three main members of Rho GTPases. The results of optical imaging of three kinds of BiLC RhoGTPase biosensors. The relative luminescence was calculated by the ratio of luminescent intensity of firefly luciferase (FL) at 600 nm to that of renilla luciferase (RL) at 500 nm (n  =  4, representative of 4 independent experiments). Error bars denote standard deviations. Asterisks (*) denotes samples that show a difference from the nonspecific complementation (the non-interactive GTPase-effector pairs or the effector loop mutants) with statistical significance by analysis of variance (ANOVA) (*p*≤0.01). This result indicates that the nonspecific complementation does not impede the correct interpretation of effective interactions induced by GTPase activation. WYJH (#) denotes samples that show a difference from the wild-type biosensor with statistical significance by ANOVA (*p*≤0.01). This result indicates that the BiLC sensors possess the discriminatory power among different GTPase activity states. (B) The results of coimmunoprecipitation. The results show that the expressions of the BiLC biosensors had no significant discrimination among different alleles of Rho biosensor, but the intensities of the PPIs displayed obvious diversities, which were in accordance with the results obtained by our optical imaging. (C) In vivo optical CCD imaging of BiLC Rho GTPase biosensors. The pseudotumors in living mice were generated by engrafting with transiently transfected 293 cells. The pRL-tk plasmid was cotransfected and RL activity was detected to normalize the planted cell number. 24 h after implantation, the mice were imaged using IVIS spectrum. A significant discrimination of luciferase activity was detected among different alleles of Rho GTPase. (I: the dominant active mutants; II: the wild-type Rho GTPases; III: the effector-loop mutants; IV: the dominant negative mutants).

**Fig 6:**
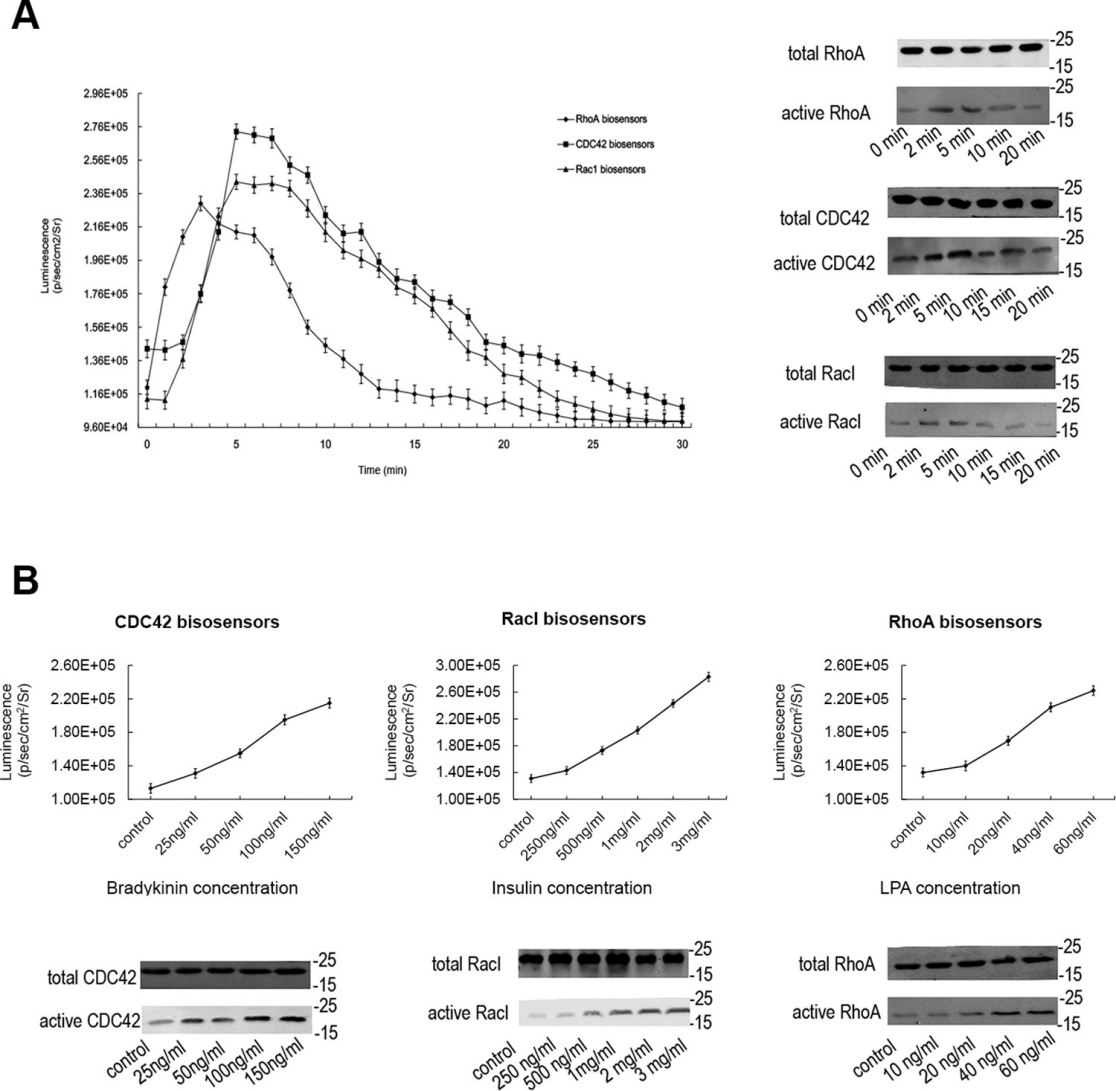
The sensitivity analysis of BiLC Rho GTPase sensors to extracellular ligands. (A) The temporal response of BiLC Rho GTPase sensors stimulated by extracellular ligands. After being serum-starved in serum-free DMEM medium for 6 h, mouse fibroblast NIH3T3 cells were detected the luminescent signals by adding D-luciferin until the intensities became steady, then stimulated with insulin (2 mg/mL), lysophosphatidic acid (40 ng/mL) and bradykinin (100 ng/mL), which are the known activator of Rac1, RhoA and Cdc42 respectively, and then immediately acquired the sequence image (1-min exposure; emission filter, open; f-stop, 1; binning, 8; field of view, 15 cm) for 30 min using IVIS spectrum. The data shown were obtained by three separate experiments performed with quadruplicate culture wells. The result shows that not only the activation signals of Rho GTPases from upstream pathways but also the subsequent decrease following the hydrolysis of GTP can be displayed and quantified by the BiLC-based biosensors. And the optical results (left) accord with that of ‘pull-down’ in our previous work (right). (B) The responses of BiLC Rho GTPase sensors to different concentration of extracellular ligands. The cells were stimulated with different concentrations of the stimulators and the luciferase activity was acquired after 3 min by IVIS spectrum (1-min exposure; emission filter, open; f-stop, 1; binning, 8; field of view, 15 cm). The data shown was obtained by three separate experiments performed with quadruplicate culture wells. The optical results (upper) were in accordance with that of our previous ‘pull-down’ (under).

The corresponding author provided the original underlying data for the blots presented in Figs 4B and 6A, published in the S1 and S2 Files available with this notice, as well as the updated Figs 4B and 6A below.

The authors did not comment on the availability of the remaining data presented in this study.

The provided data and updated figures support the findings reported in [1], but the *PLOS ONE* Editors remain concerned about the number of panels affected by data reporting issues. We issue this Expression of Concern to notify readers of the above concerns and to relay the available data provided by the corresponding author.

## Supporting information

S1 File Original blots underlying Fig 4B results. (ZIP)

S2 File Original blots underlying Fig 6A results. (ZIP)

S3 File Comparison underlying data and published panels for Figs 4B and 6A. (ZIP)

## References

[1] Leng W, Pang X, Xia H, Li M, Chen L, Tang Q, et al. (2013) Novel Split-Luciferase-Based Genetically Encoded Biosensors for Noninvasive Visualization of Rho GTPases. PLoS ONE 8(4): e62230. 10.1371/journal.pone.0062230 23614039 PMC3627919

