# Supplementary figures and images for: Expression of Concern: Novel Split-Luciferase-Based Genetically Encoded Biosensors for Noninvasive Visualization of Rho GTPases
Source: PLoS One. 2023 Jun 23;18(6):e0287871. doi: 10.1371/journal.pone.0287871 (PMC10289462; doi:10.1371/journal.pone.0287871)

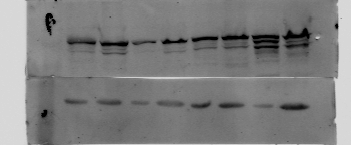

Supplement: S2 File — (ZIP) [file pone.0287871.s002.zip › S2 File Underlying data Figure 6A/1sehj.tif]
