# Supplementary material for: Expression of Concern: Novel Split-Luciferase-Based Genetically Encoded Biosensors for Noninvasive Visualization of Rho GTPases
Source: PLoS One. 2023 Jun 23;18(6):e0287871. doi: 10.1371/journal.pone.0287871 (PMC10289462; doi:10.1371/journal.pone.0287871)
Supplement: S3 File — (ZIP) [file pone.0287871.s003.zip › S3 File - comparison underlying data and corrected panels/All correct and updated panels for Fig4B.pptx]

## Slide 1
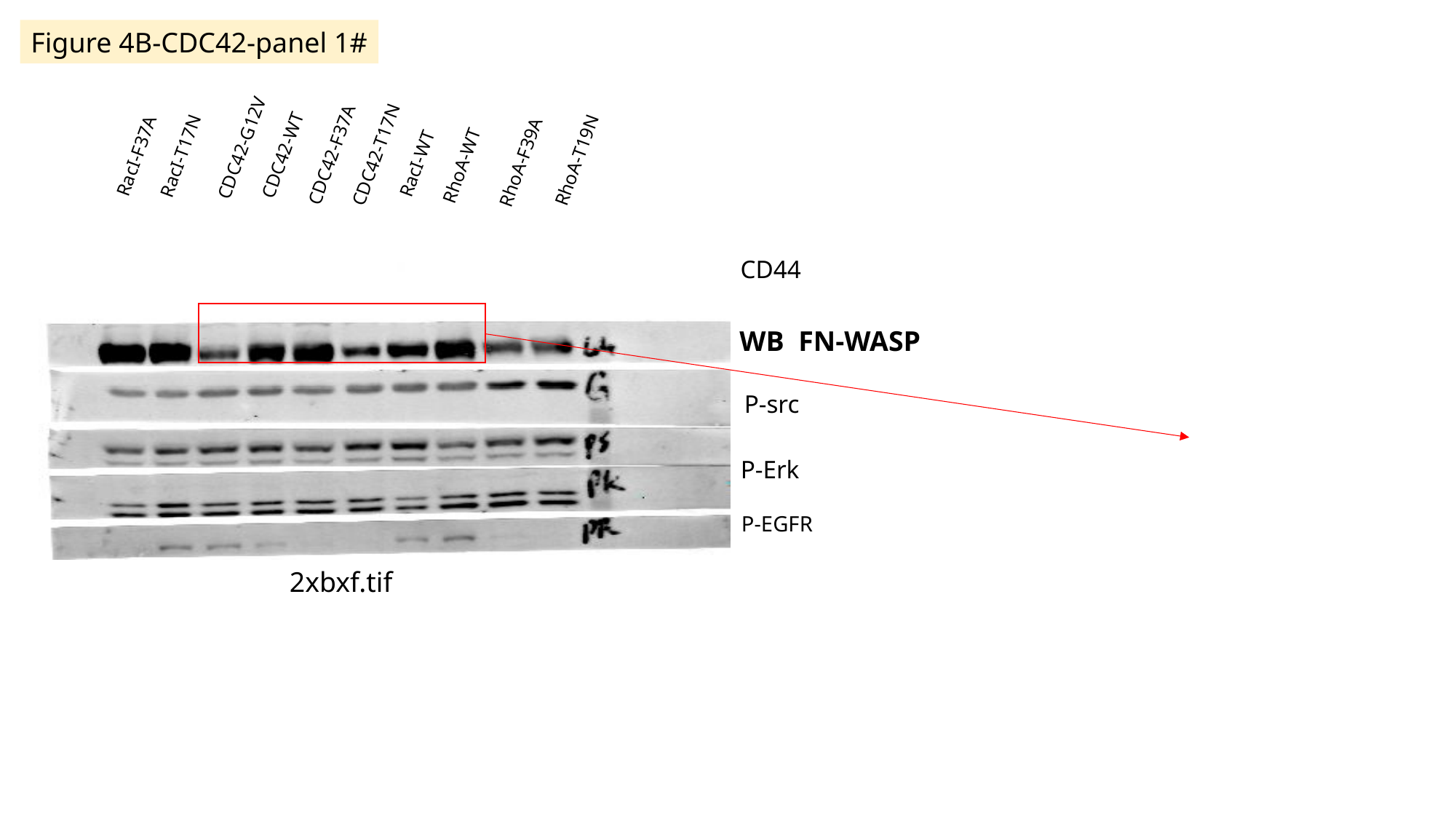

Figure 4B-CDC42-panel 1#
CDC42-G12V
CDC42-T17N
CDC42-F37A
CDC42-WT
RhoA-WT
RacI-WT
RhoA-T19N
RacI-T17N
RacI-F37A
RhoA-F39A
CD44
WB FN-WASP
P-src
P-Erk
P-EGFR
2xbxf.tif

## Slide 2
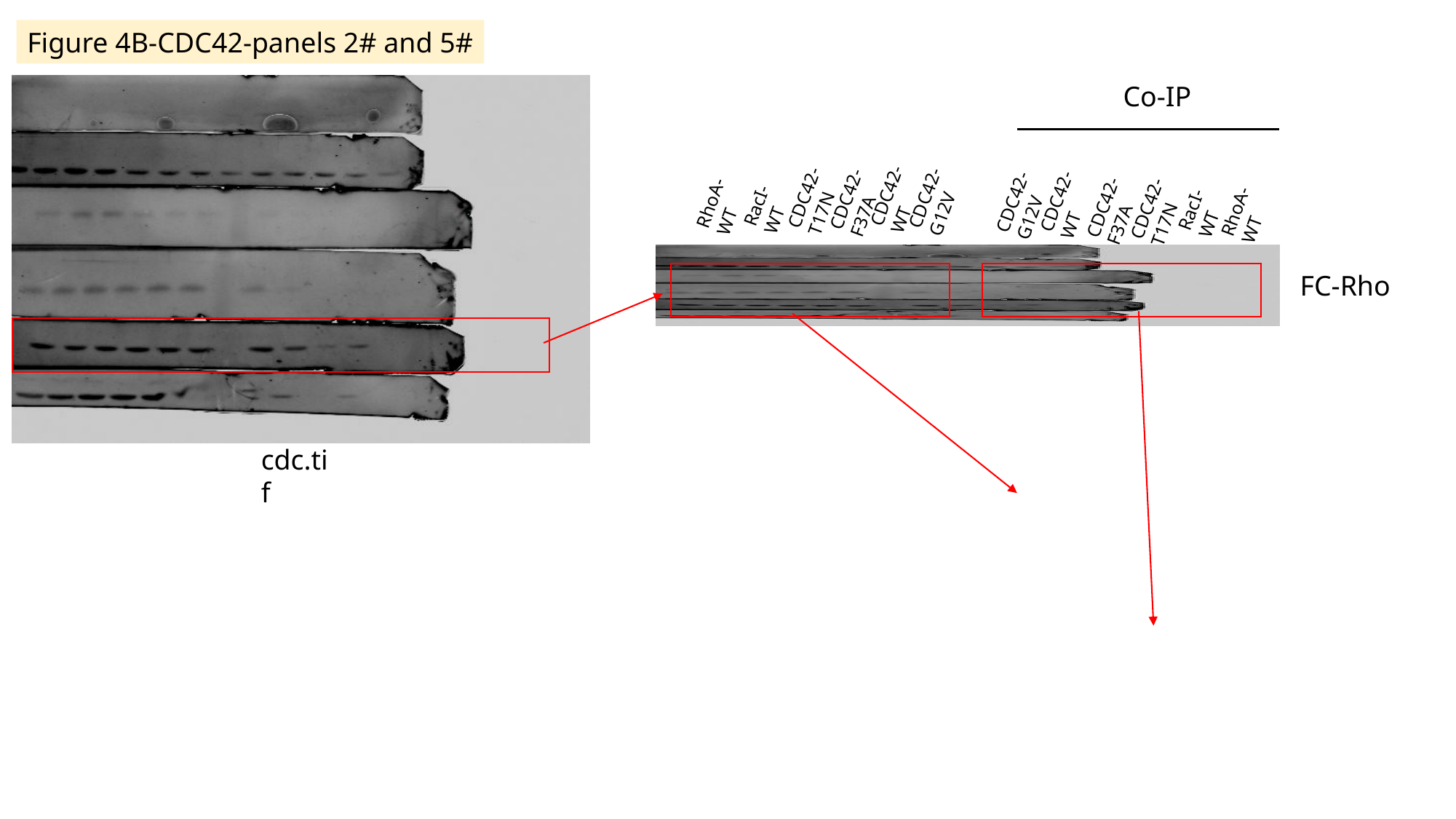

Figure 4B-CDC42-panels 2# and 5#
Co-IP
CDC42-G12V
CDC42-T17N
CDC42-G12V
CDC42-F37A
CDC42-T17N
CDC42-F37A
CDC42-WT
CDC42-WT
RhoA-WT
RhoA-WT
RacI-WT
RacI-WT
FC-Rho
cdc.tif

## Slide 3
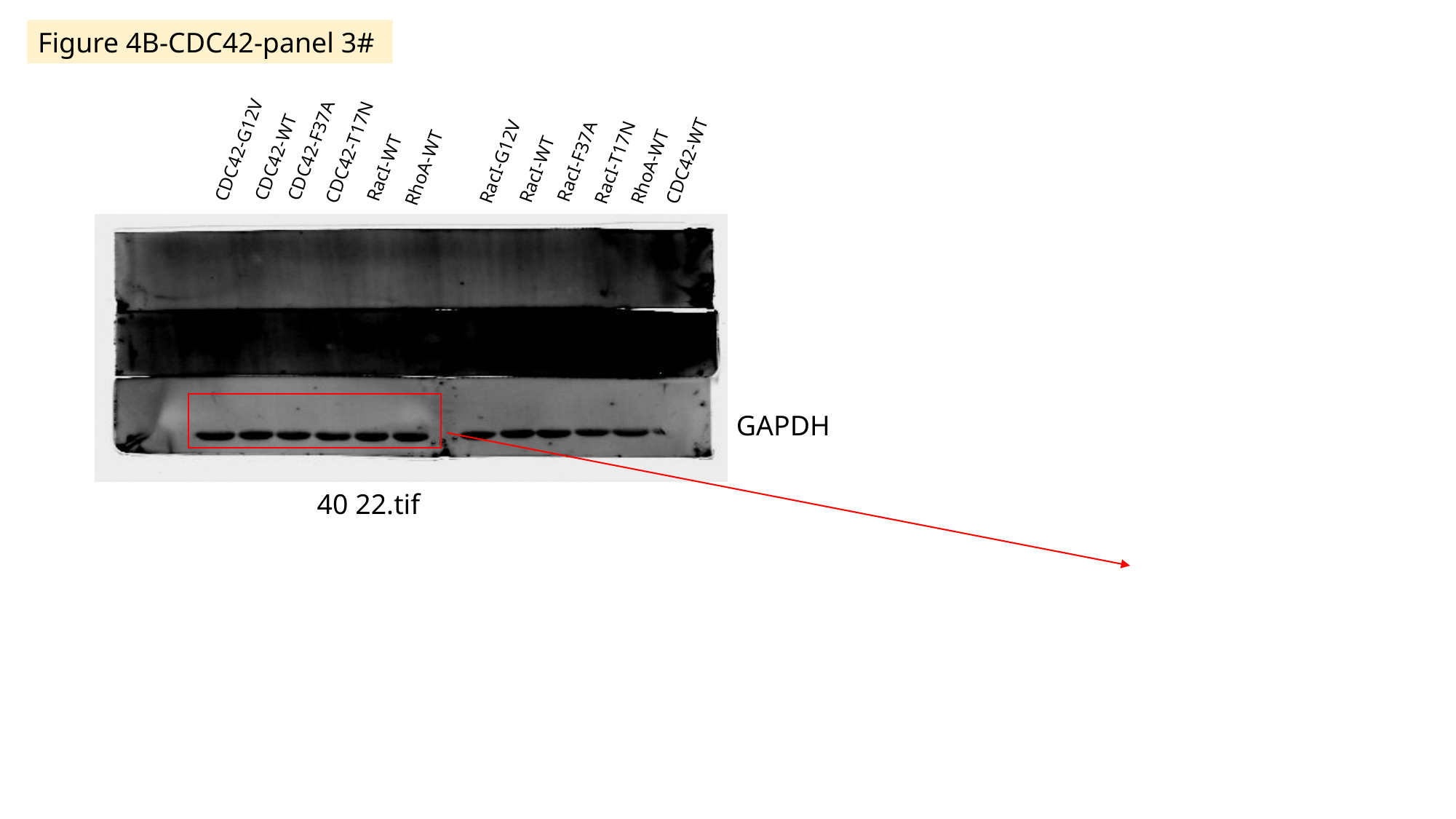

Figure 4B-CDC42-panel 3#
CDC42-G12V
CDC42-T17N
CDC42-F37A
CDC42-WT
RhoA-WT
RacI-WT
CDC42-WT
RacI-G12V
RacI-T17N
RacI-F37A
RhoA-WT
RacI-WT
GAPDH
40 22.tif

## Slide 4
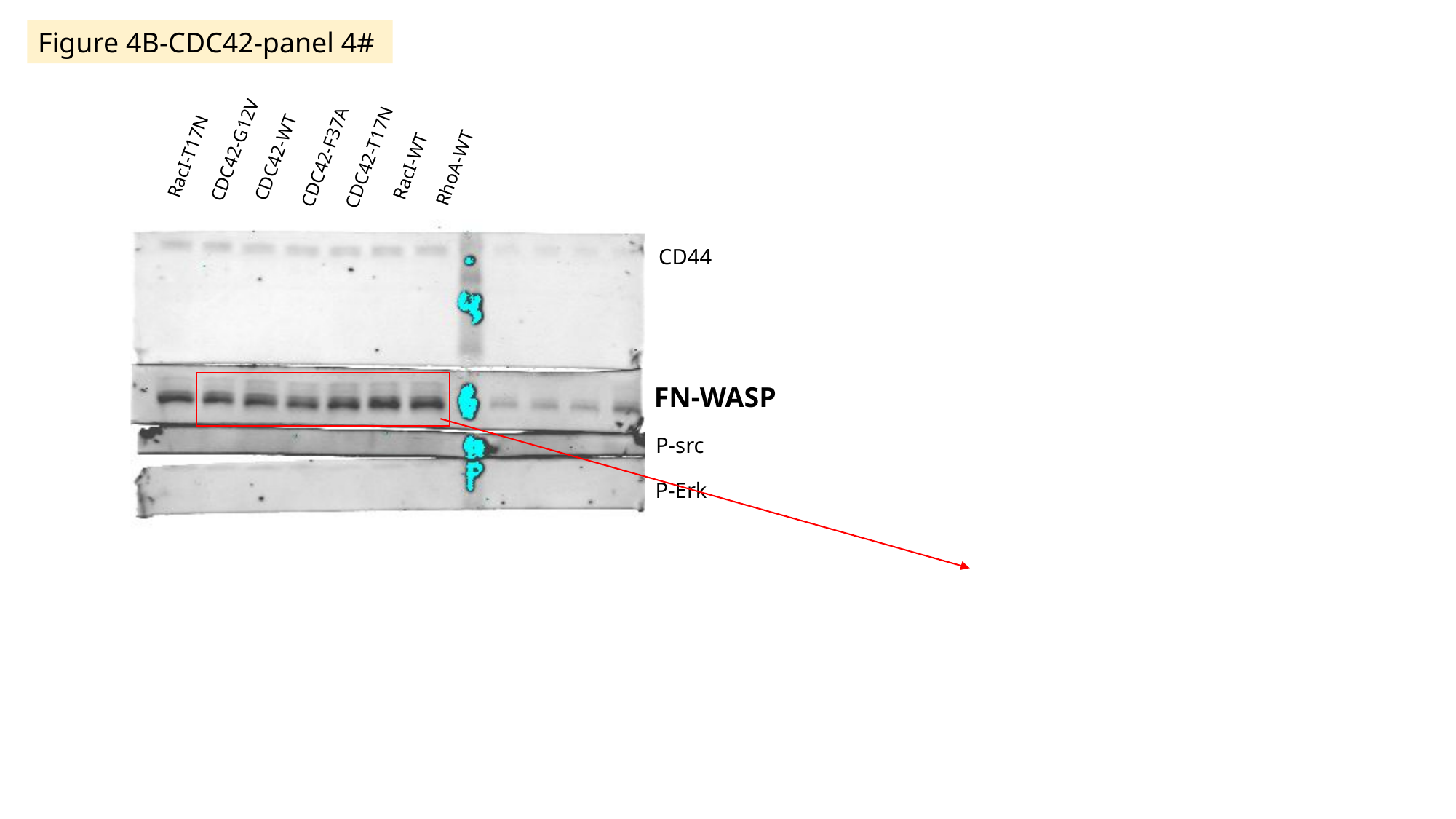

Figure 4B-CDC42-panel 4#
CDC42-G12V
CDC42-T17N
CDC42-F37A
CDC42-WT
RhoA-WT
RacI-WT
RacI-T17N
CD44
FN-WASP
P-src
P-Erk

## Slide 5
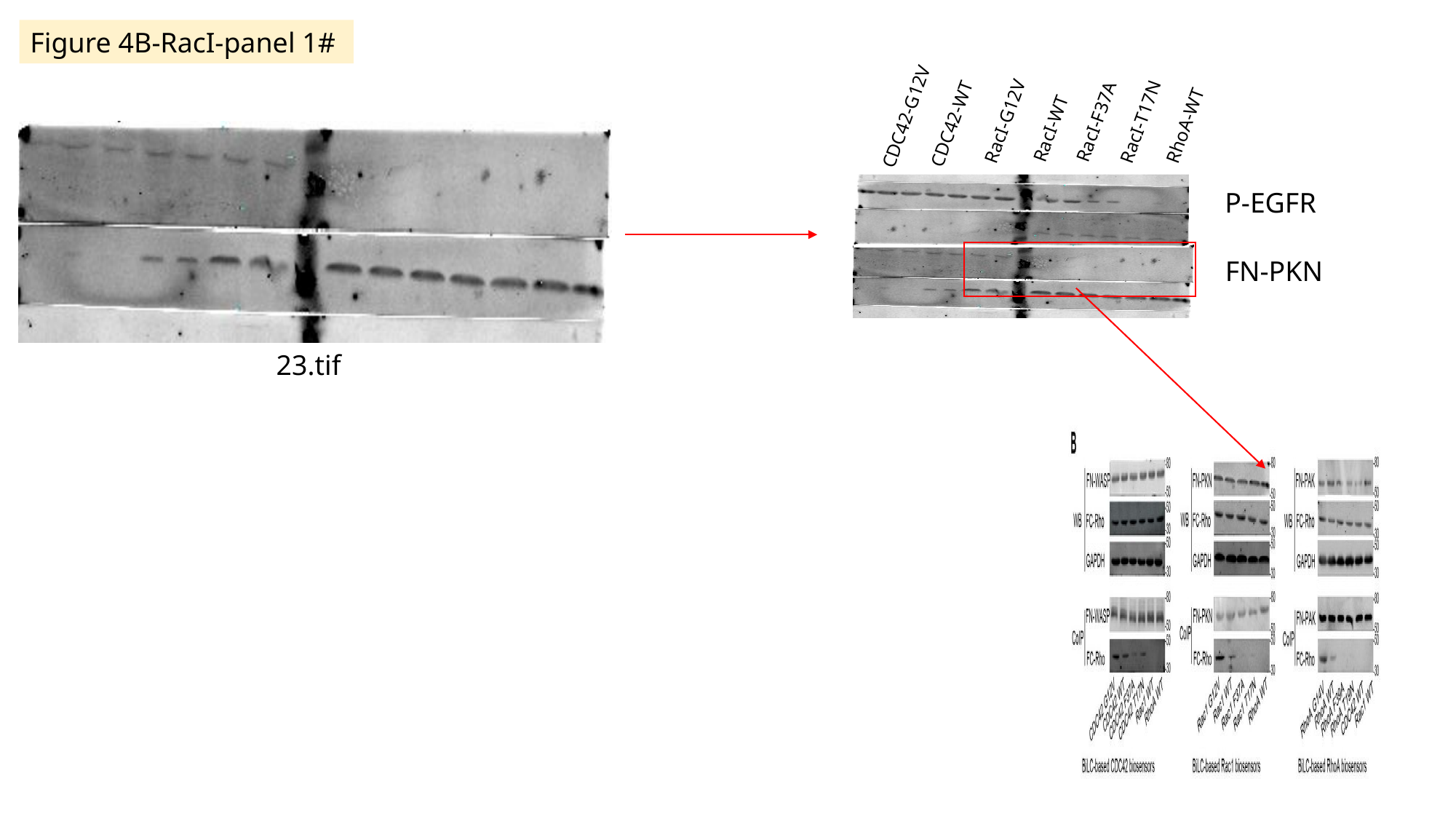

Figure 4B-RacI-panel 1#
CDC42-G12V
CDC42-WT
RacI-G12V
RacI-T17N
RacI-F37A
RhoA-WT
RacI-WT
23.tif
P-EGFR
FN-PKN

## Slide 6
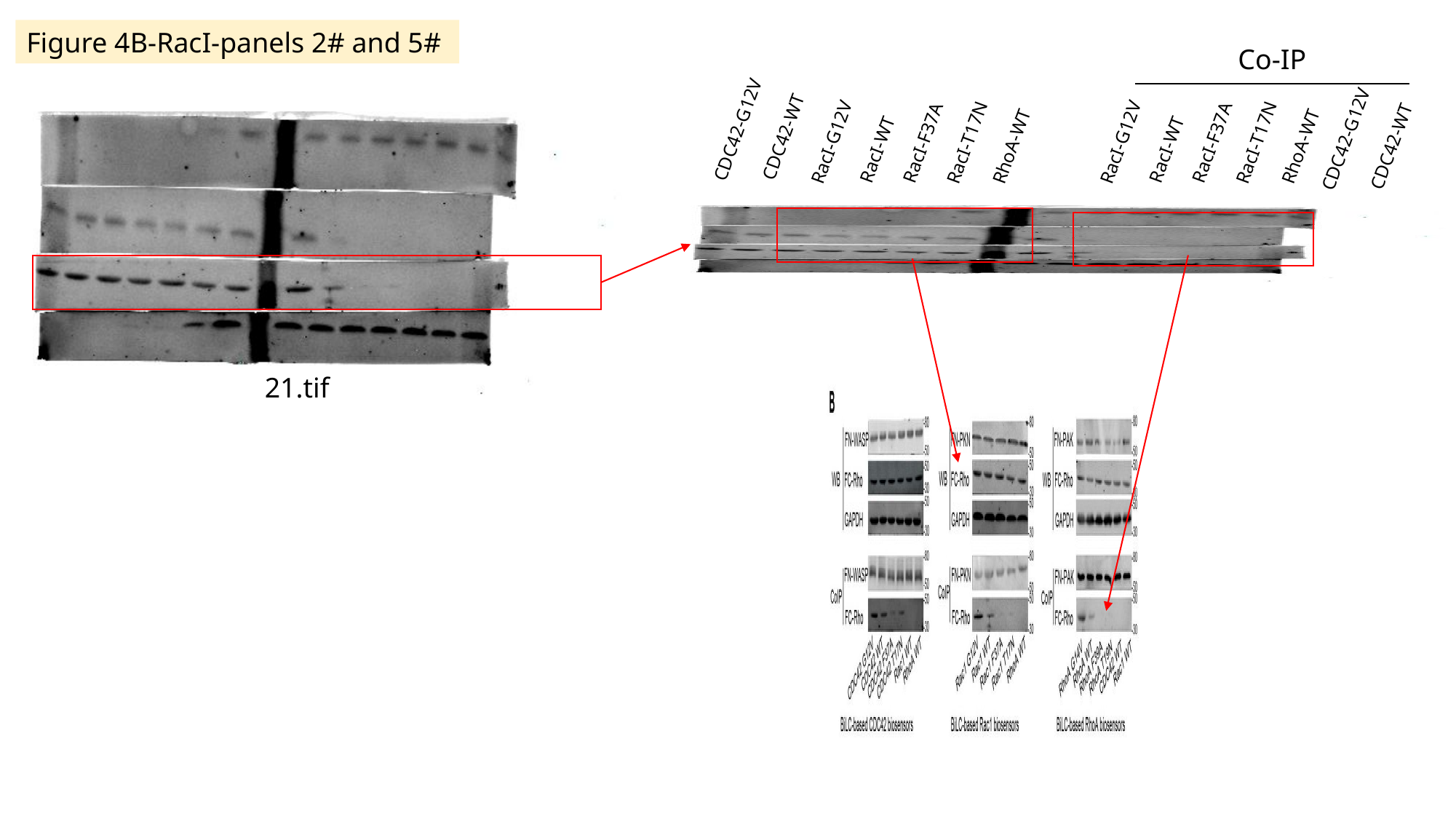

Figure 4B-RacI-panels 2# and 5#
Co-IP
CDC42-G12V
CDC42-WT
RacI-G12V
RacI-T17N
RacI-F37A
RhoA-WT
RacI-WT
CDC42-G12V
CDC42-WT
RacI-G12V
RacI-T17N
RacI-F37A
RhoA-WT
RacI-WT
21.tif

## Slide 7
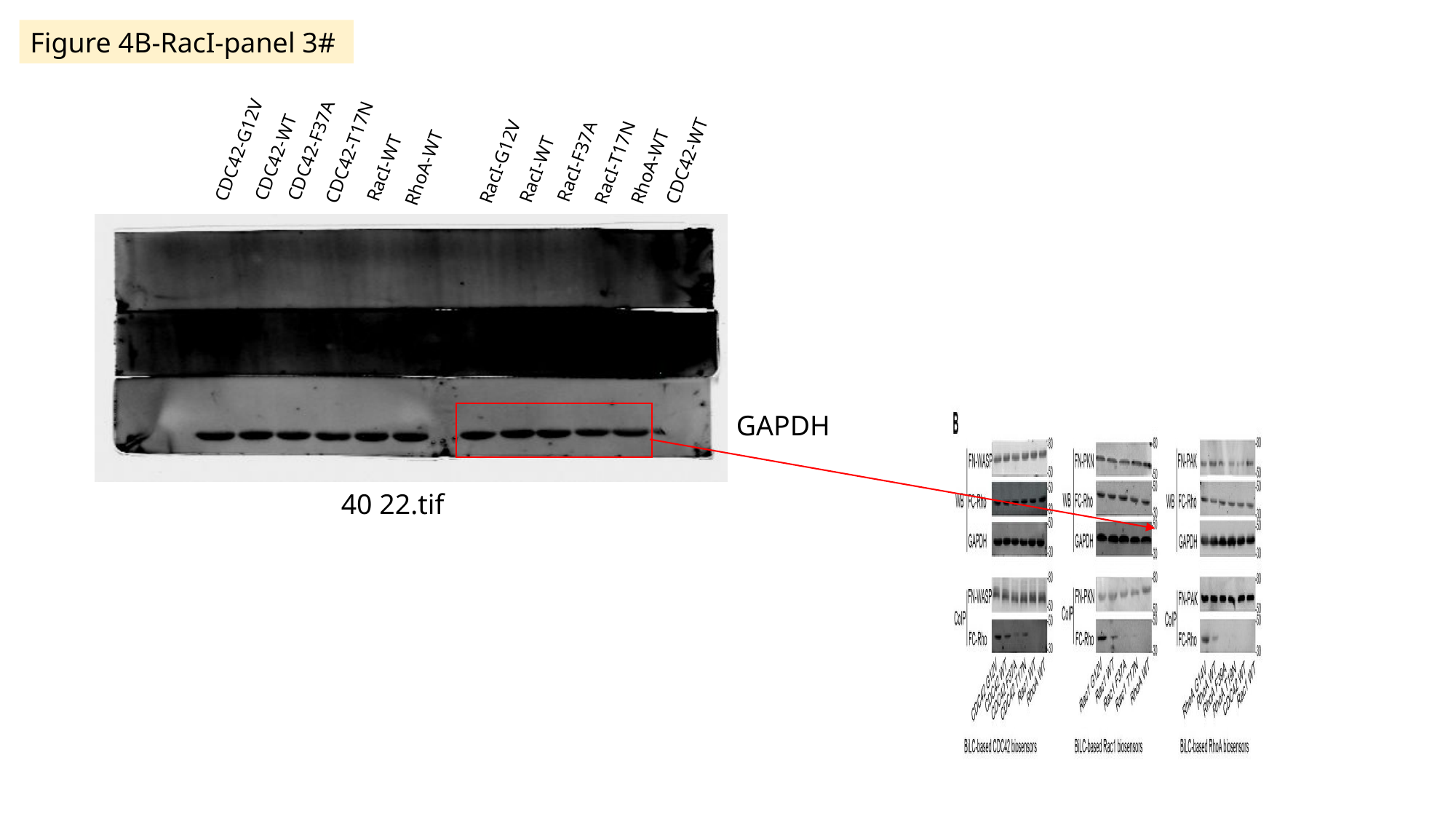

Figure 4B-RacI-panel 3#
CDC42-G12V
CDC42-T17N
CDC42-F37A
CDC42-WT
RhoA-WT
RacI-WT
CDC42-WT
RacI-G12V
RacI-T17N
RacI-F37A
RhoA-WT
RacI-WT
GAPDH
40 22.tif

## Slide 8
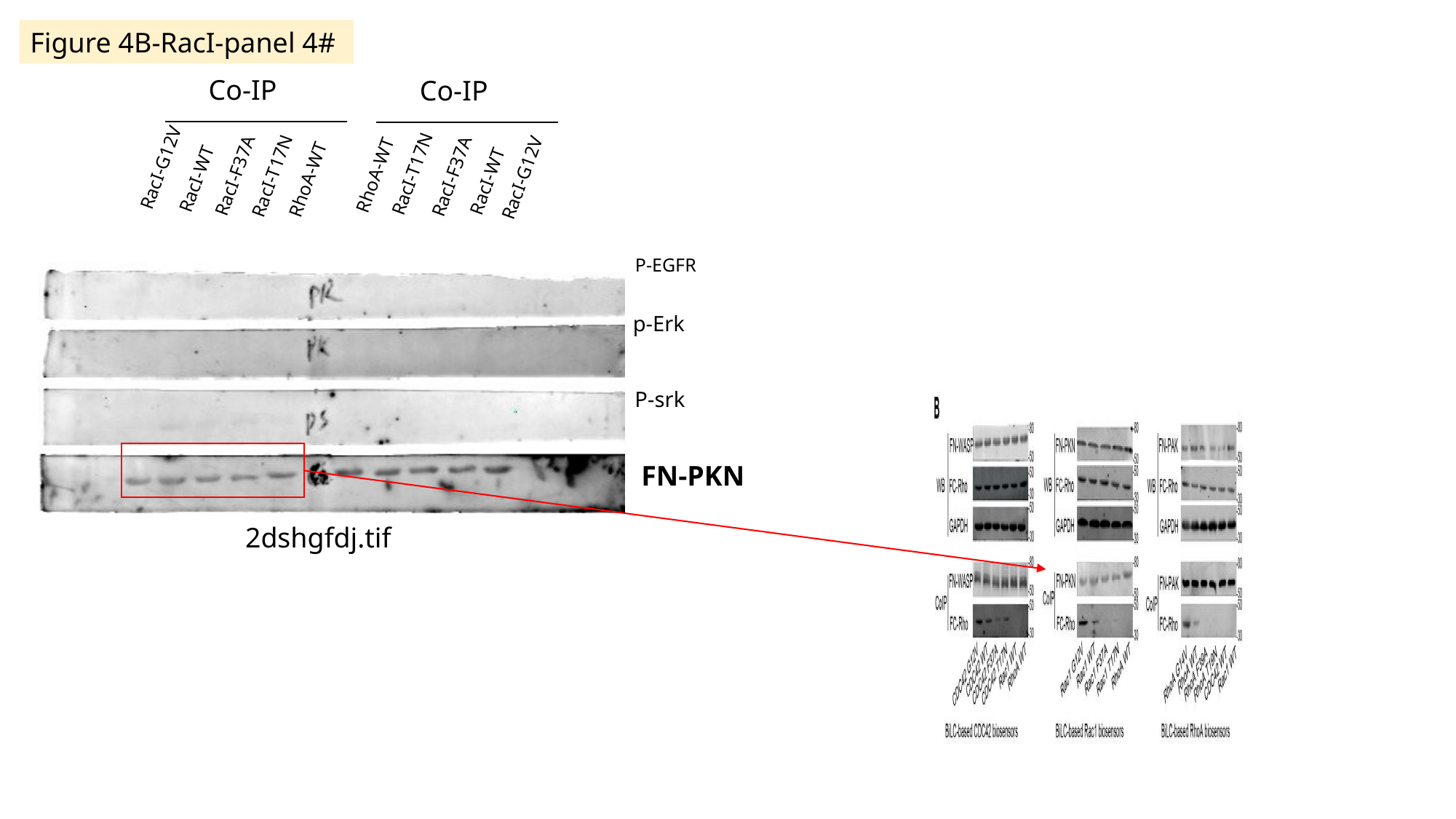

Figure 4B-RacI-panel 4#
Co-IP
RacI-G12V
RacI-T17N
RacI-F37A
RhoA-WT
RacI-WT
Co-IP
RacI-T17N
RhoA-WT
RacI-G12V
RacI-F37A
RacI-WT
P-EGFR
p-Erk
P-srk
FN-PKN
2dshgfdj.tif

## Slide 9
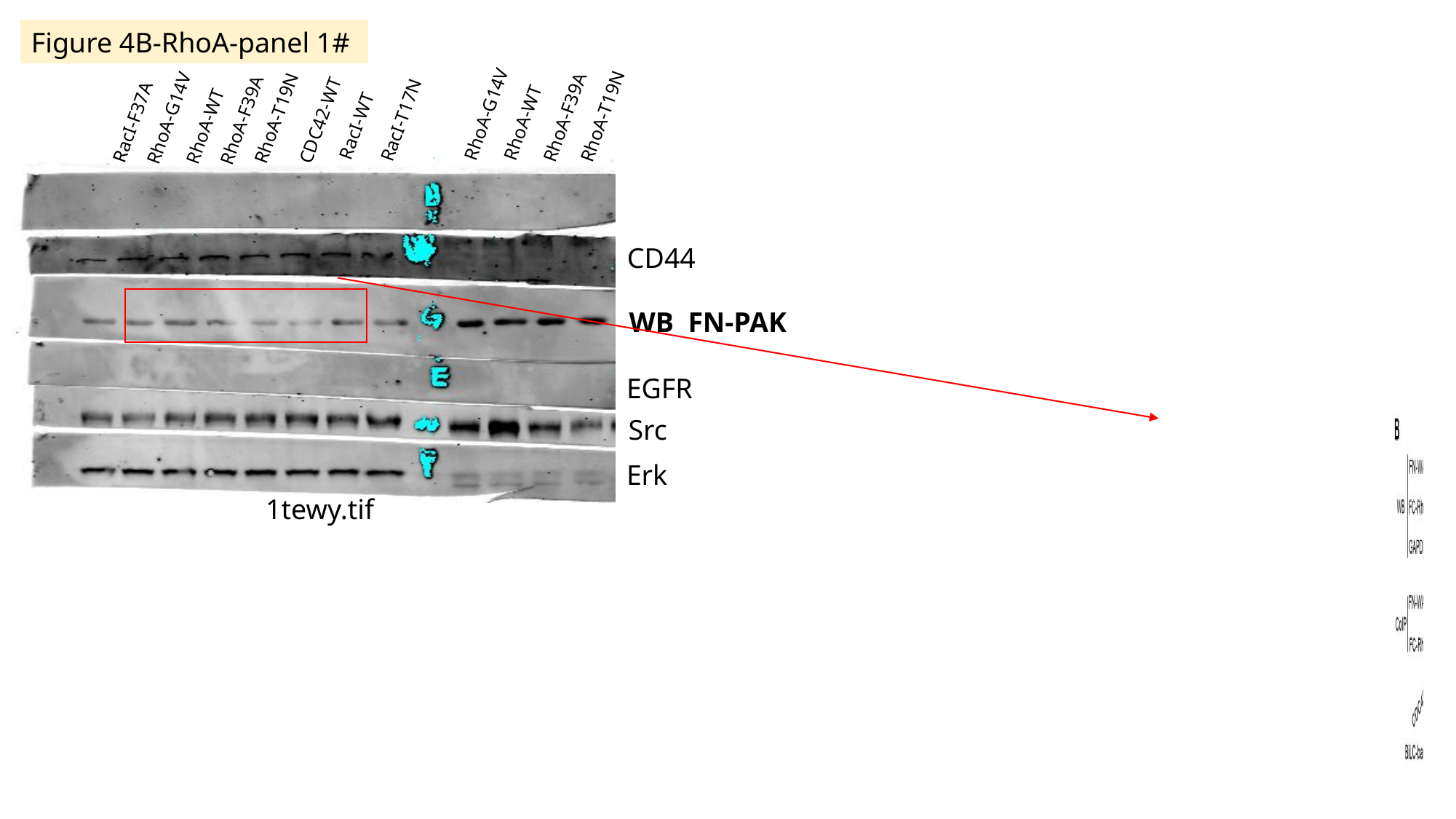

Figure 4B-RhoA-panel 1#
RhoA-G14V
RhoA-T19N
RhoA-F39A
RhoA-WT
RhoA-G14V
RhoA-T19N
RhoA-F39A
CDC42-WT
RacI-T17N
RhoA-WT
RacI-WT
RacI-F37A
CD44
WB FN-PAK
EGFR
Src
Erk
1tewy.tif

## Slide 10
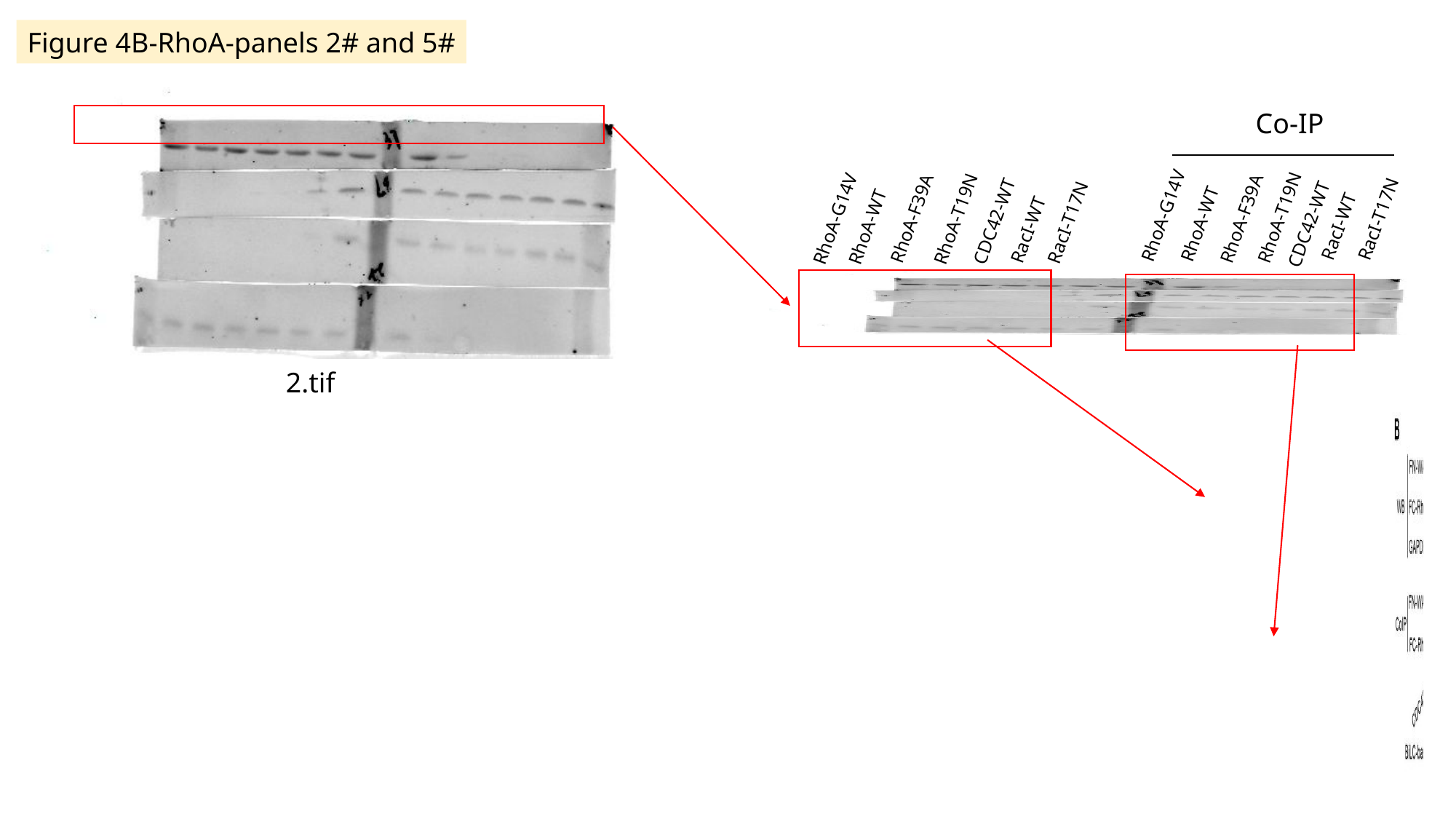

Figure 4B-RhoA-panels 2# and 5#
Co-IP
RhoA-G14V
RhoA-T19N
RhoA-F39A
RacI-T17N
CDC42-WT
RhoA-WT
RacI-WT
RhoA-G14V
RhoA-T19N
RhoA-F39A
CDC42-WT
RacI-T17N
RhoA-WT
RacI-WT
2.tif

## Slide 11
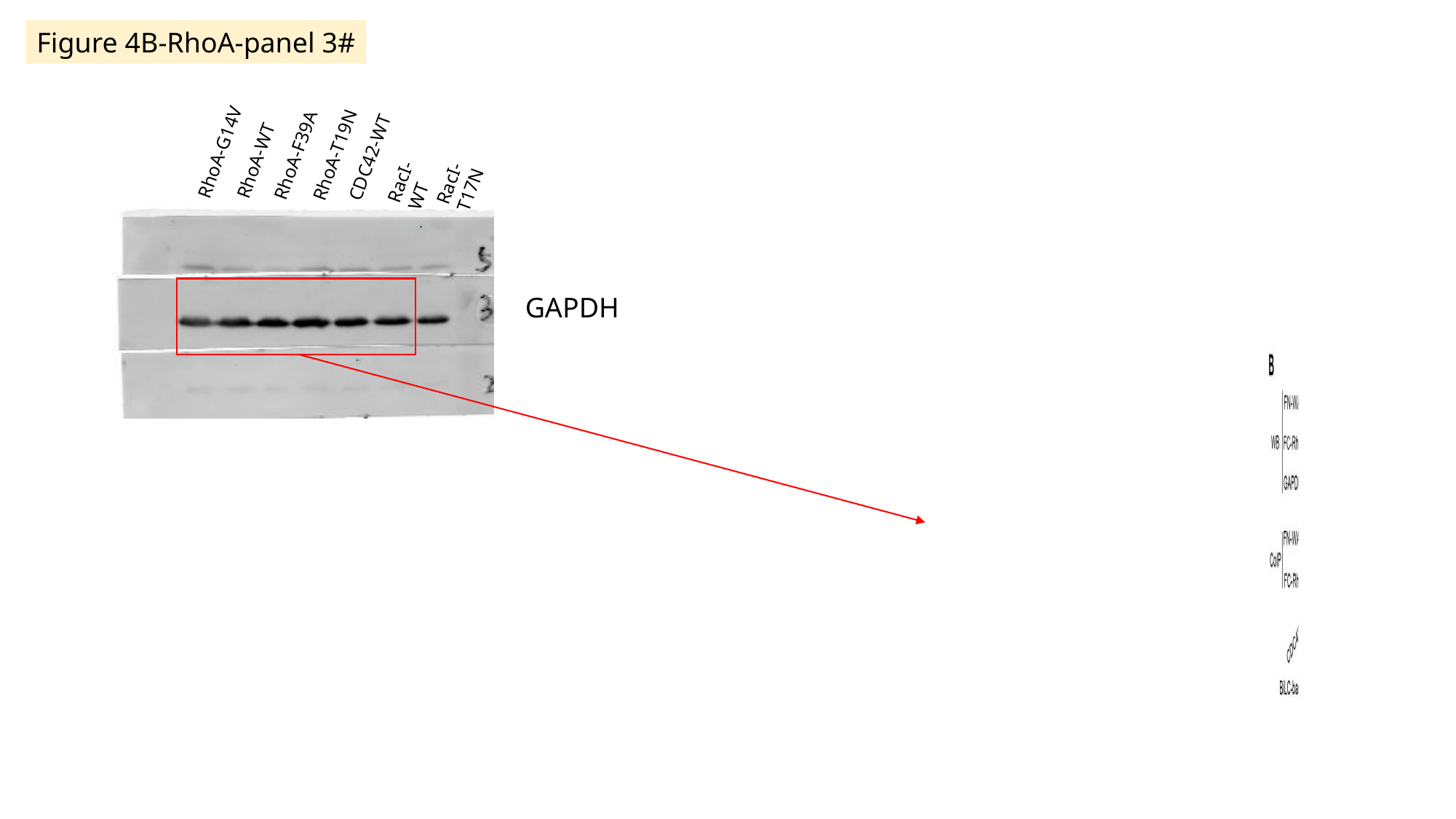

Figure 4B-RhoA-panel 3#
RhoA-G14V
RhoA-T19N
RhoA-F39A
CDC42-WT
RacI-T17N
RhoA-WT
RacI-WT
GAPDH

## Slide 12
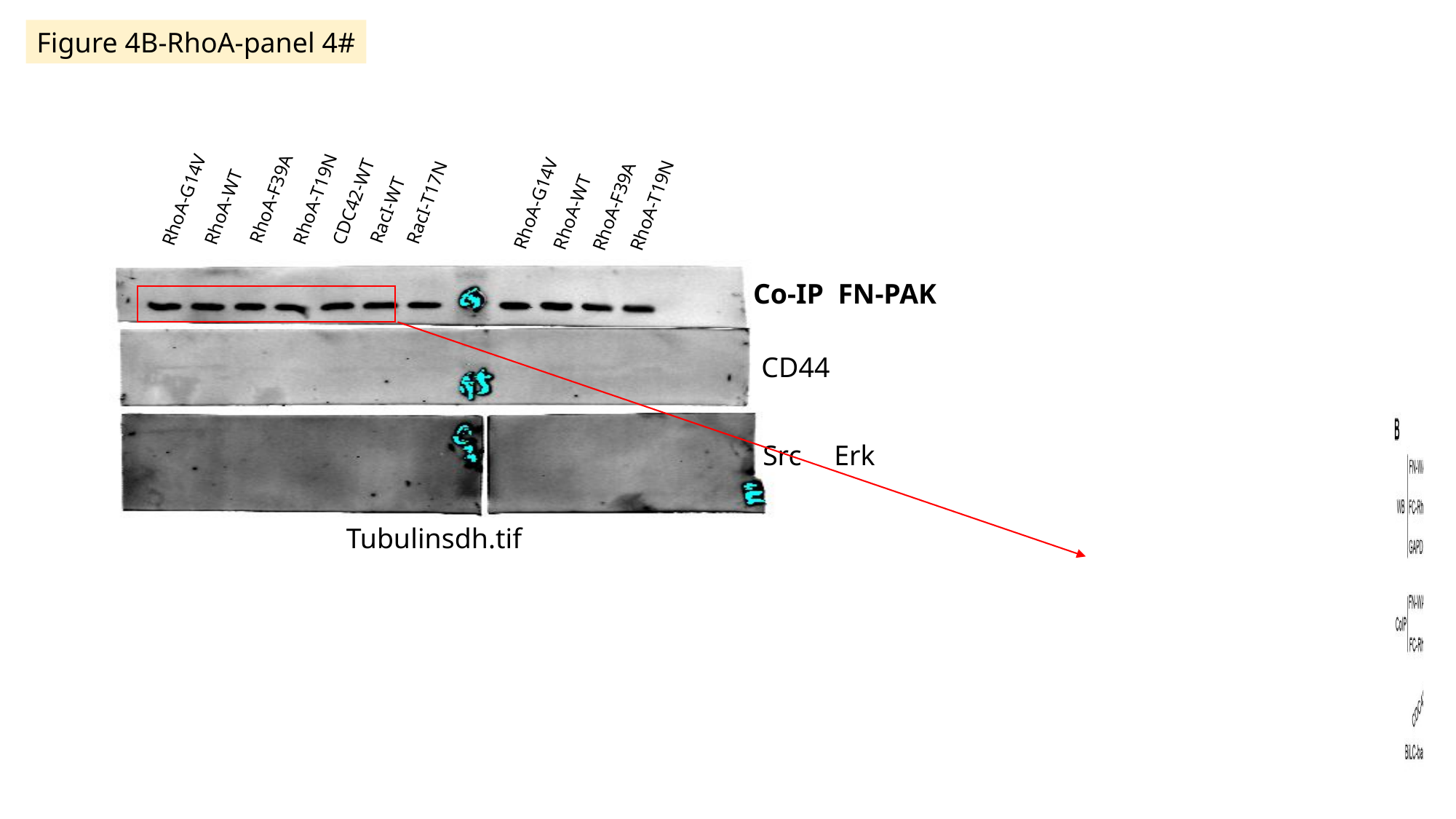

Figure 4B-RhoA-panel 4#
RhoA-T19N
RhoA-G14V
RhoA-F39A
CDC42-WT
RacI-T17N
RhoA-WT
RacI-WT
RhoA-G14V
RhoA-T19N
RhoA-F39A
RhoA-WT
Tubulinsdh.tif
Co-IP FN-PAK
CD44
Src
Erk
