# Supplementary material for: Expression of Concern: Novel Split-Luciferase-Based Genetically Encoded Biosensors for Noninvasive Visualization of Rho GTPases
Source: PLoS One. 2023 Jun 23;18(6):e0287871. doi: 10.1371/journal.pone.0287871 (PMC10289462; doi:10.1371/journal.pone.0287871)
Supplement: S3 File — (ZIP) [file pone.0287871.s003.zip › S3 File - comparison underlying data and corrected panels/All correct and updated panels for Fig6A.pptx]

## Slide 1
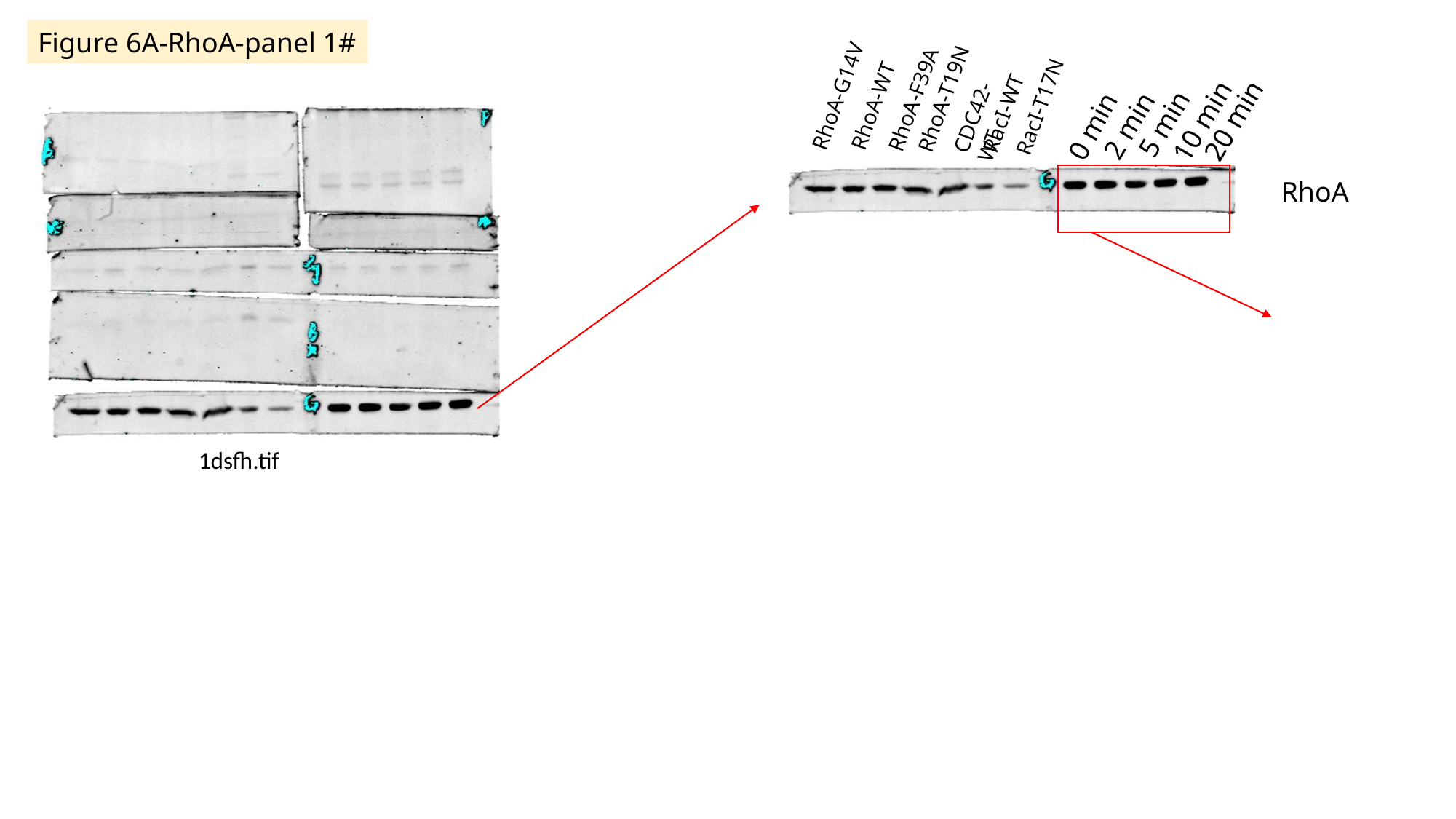

Figure 6A-RhoA-panel 1#
RhoA-F39A
RhoA-T19N
RhoA-G14V
RacI-T17N
RhoA-WT
CDC42-WT
RacI-WT
5 min
2 min
0 min
20 min
10 min
RhoA
1dsfh.tif

## Slide 2
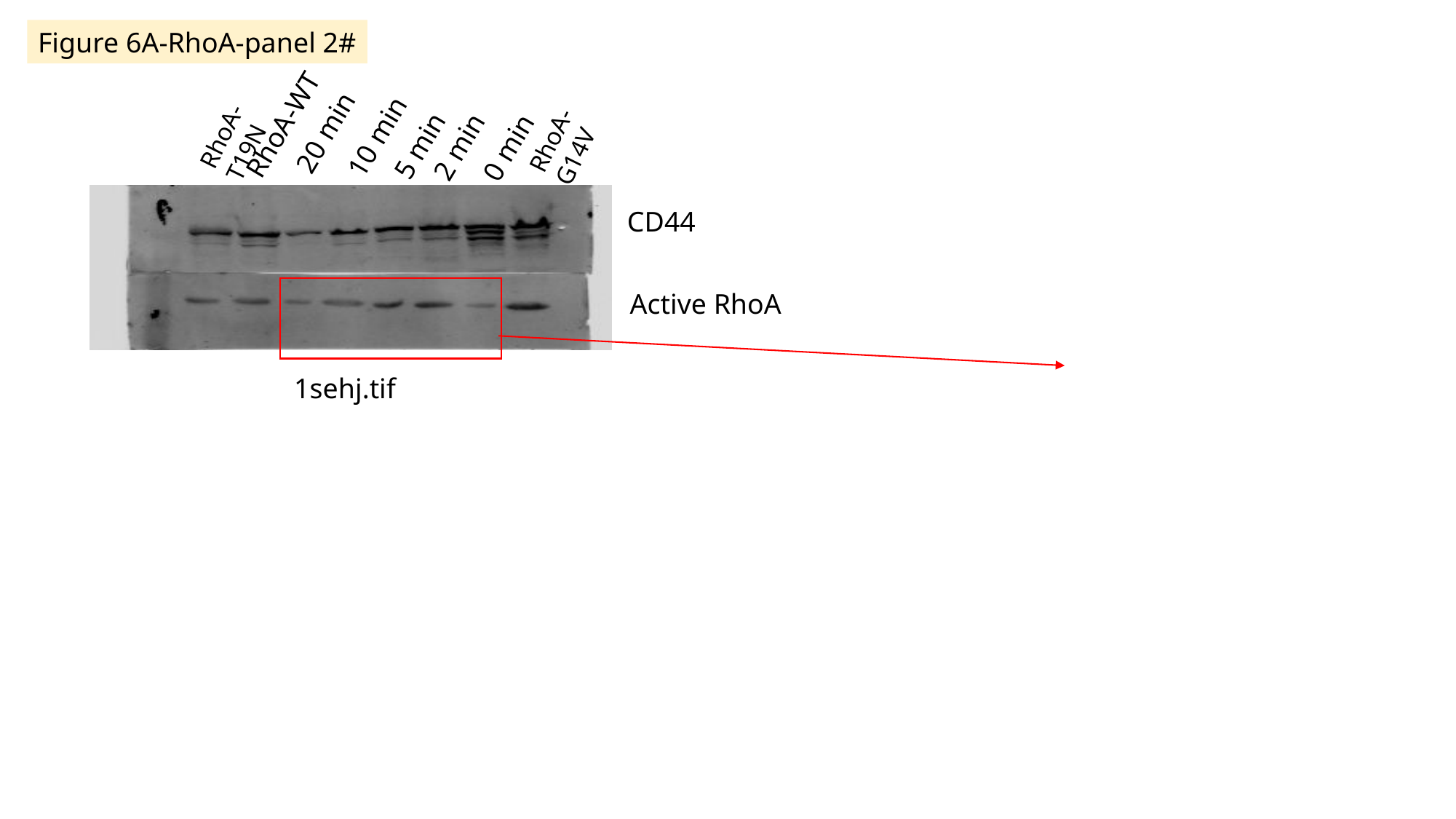

Figure 6A-RhoA-panel 2#
RhoA-T19N
RhoA-G14V
RhoA-WT
20 min
10 min
5 min
2 min
0 min
CD44
Active RhoA
1sehj.tif

## Slide 3
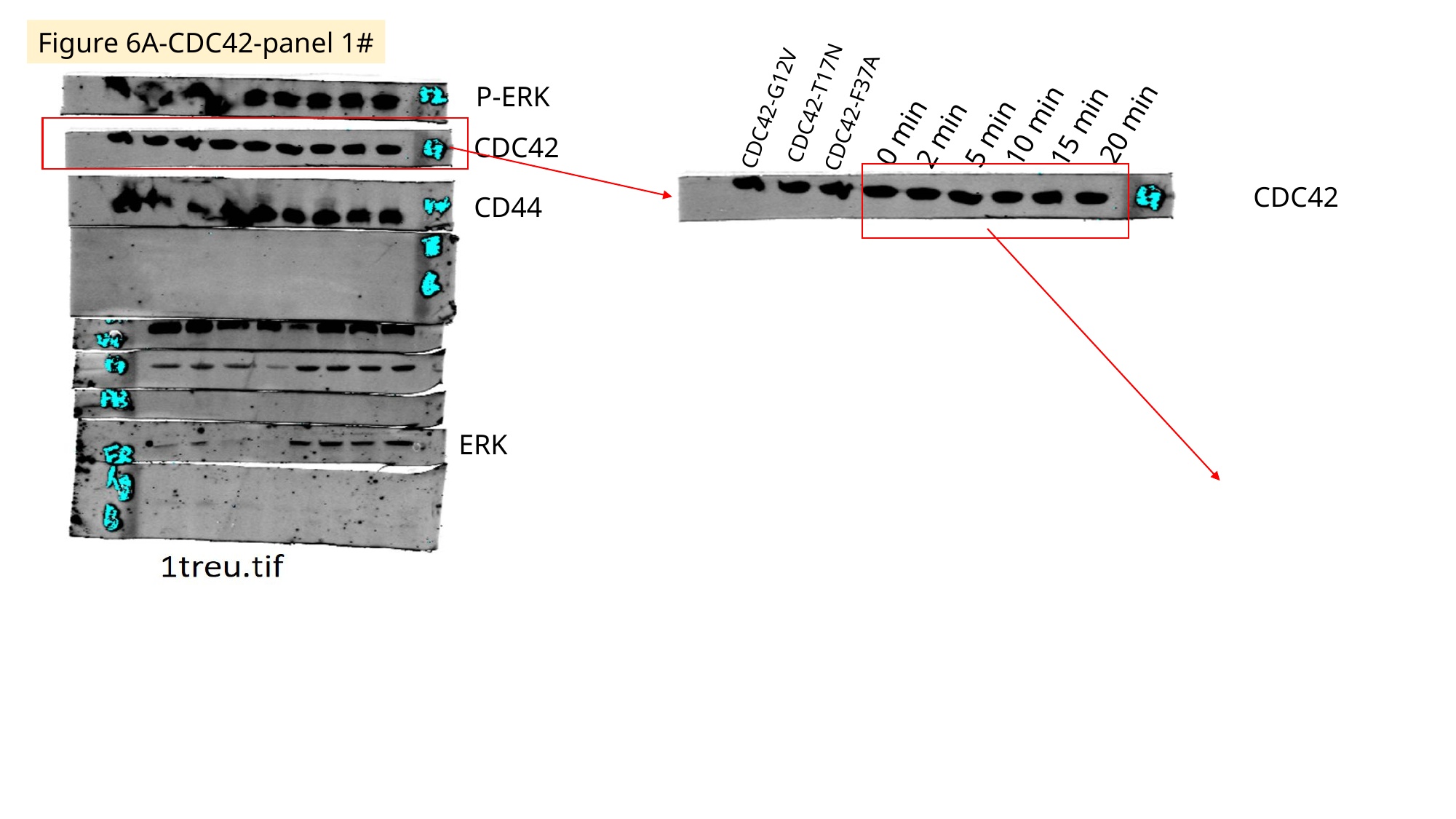

Figure 6A-CDC42-panel 1#
CDC42-T17N
CDC42-G12V
CDC42-F37A
20 min
10 min
0 min
15 min
5 min
2 min
P-ERK
CDC42
CDC42
CD44
ERK

## Slide 4
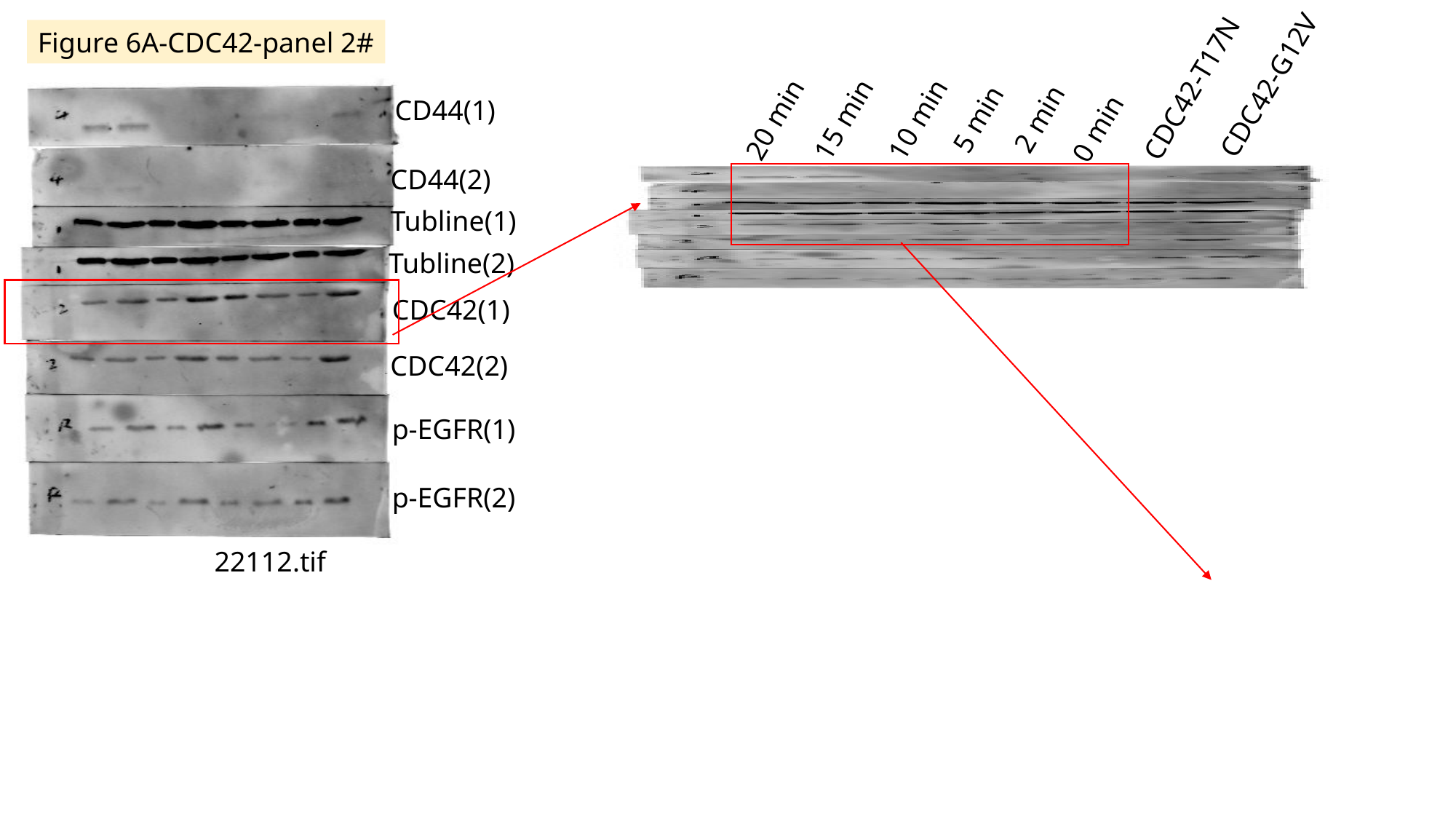

Figure 6A-CDC42-panel 2#
CDC42-G12V
CDC42-T17N
5 min
2 min
CD44(1)
20 min
10 min
15 min
0 min
CD44(2)
Tubline(1)
Tubline(2)
CDC42(1)
CDC42(2)
p-EGFR(1)
p-EGFR(2)
22112.tif

## Slide 5
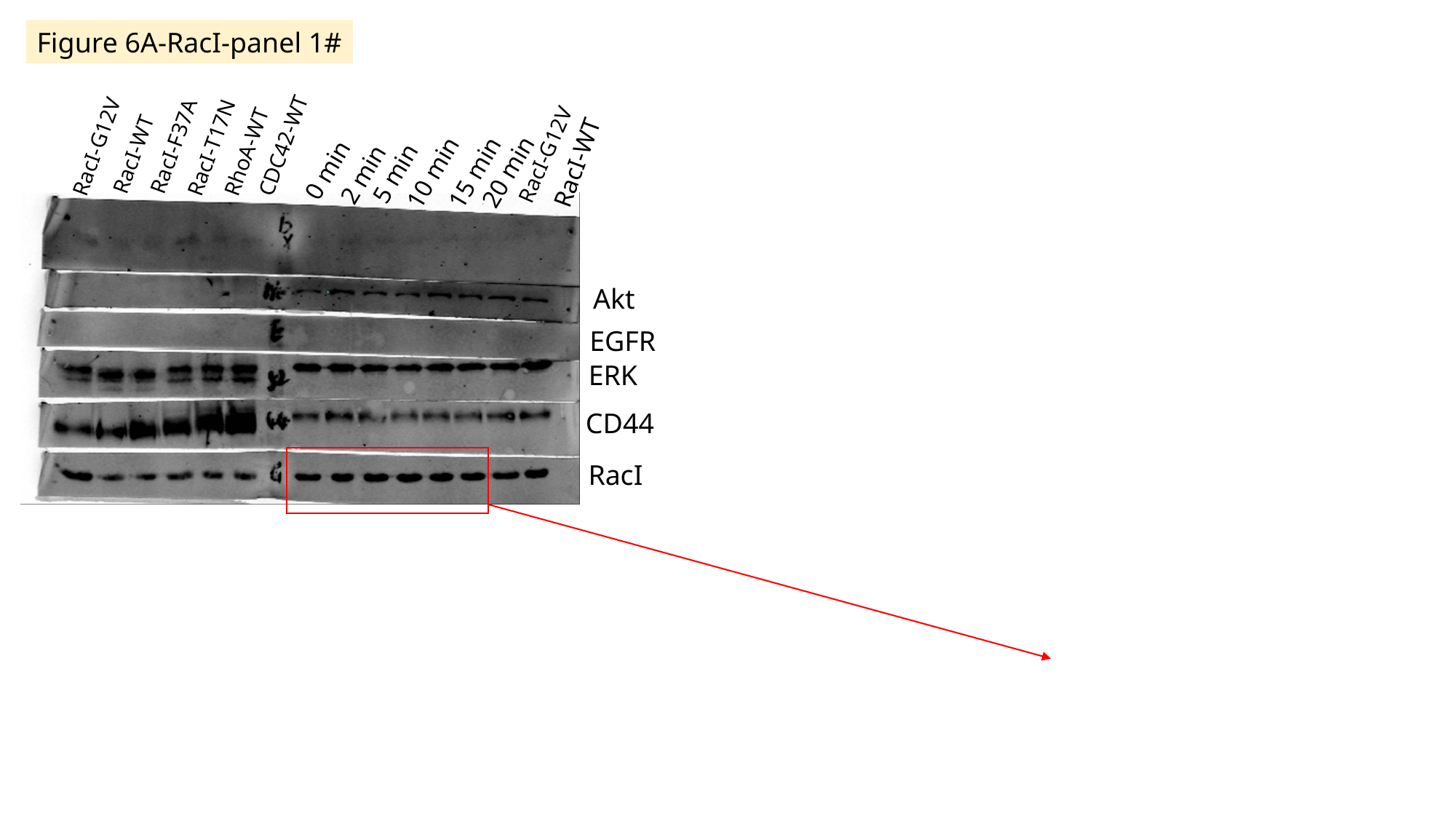

Figure 6A-RacI-panel 1#
RacI-G12V
0 min
5 min
2 min
20 min
10 min
15 min
CDC42-WT
RacI-G12V
RacI-T17N
RacI-F37A
RhoA-WT
RacI-WT
RacI-WT
Akt
EGFR
ERK
CD44
RacI

## Slide 6
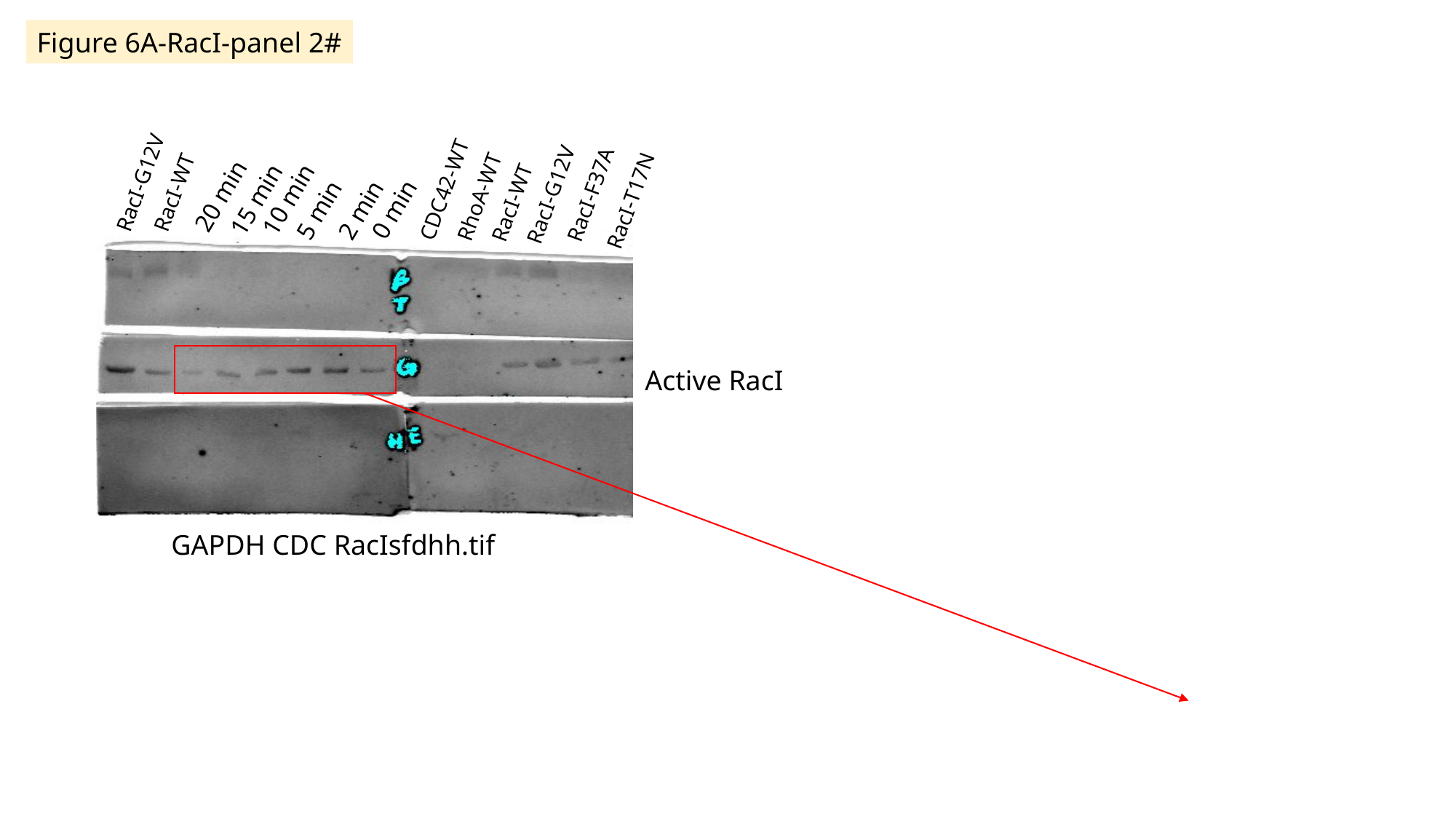

Figure 6A-RacI-panel 2#
20 min
10 min
15 min
0 min
5 min
2 min
RacI-G12V
CDC42-WT
RacI-G12V
RacI-F37A
RhoA-WT
RacI-T17N
RacI-WT
RacI-WT
Active RacI
GAPDH CDC RacIsfdhh.tif
